# How Thioredoxin Dissociates Its Mixed Disulfide

**DOI:** 10.1371/journal.pcbi.1000461

**Published:** 2009-08-13

**Authors:** Goedele Roos, Nicolas Foloppe, Koen Van Laer, Lode Wyns, Lennart Nilsson, Paul Geerlings, Joris Messens

**Affiliations:** 1 Department of Molecular and Cellular Interactions, Vlaams Interuniversitair Instituut voor Biotechnologie, Brussels, Belgium; 2 Structural Biology Brussels, Vrije Universiteit Brussel, Brussels, Belgium; 3 Brussels Center for Redox Biology, Brussels, Belgium; 4 Algemene Chemie, Vrije Universiteit Brussel, Brussels, Belgium; 5 Department of Biosciences and Nutrition, Karolinska Institutet, Huddinge, Sweden; Saarland University, Germany

## Abstract

The dissociation mechanism of the thioredoxin (Trx) mixed disulfide complexes is unknown and has been debated for more than twenty years. Specifically, opposing arguments for the activation of the nucleophilic cysteine as a thiolate during the dissociation of the complex have been put forward. As a key model, the complex between Trx and its endogenous substrate, arsenate reductase (ArsC), was used. In this structure, a Cys29^Trx^-Cys89^ArsC^ intermediate disulfide is formed by the nucleophilic attack of Cys29^Trx^ on the exposed Cys82^ArsC^-Cys89^ArsC^ in oxidized ArsC. With theoretical reactivity analysis, molecular dynamics simulations, and biochemical complex formation experiments with Cys-mutants, Trx mixed disulfide dissociation was studied. We observed that the conformational changes around the intermediate disulfide bring Cys32^Trx^ in contact with Cys29^Trx^. Cys32^Trx^ is activated for its nucleophilic attack by hydrogen bonds, and Cys32^Trx^ is found to be more reactive than Cys82^ArsC^. Additionally, Cys32^Trx^ directs its nucleophilic attack on the more susceptible Cys29^Trx^ and not on Cys89^ArsC^. This multidisciplinary approach provides fresh insights into a universal thiol/disulfide exchange reaction mechanism that results in reduced substrate and oxidized Trx.

## Author Summary

Thioredoxins are found in all types of cells and control several essential functions of life, including promotion of cell growth, inhibition of apoptosis, and modulation of inflammation. Thioredoxin has two ‘free’ cysteines in its active site, which are used to break disulfide bonds in oxidized substrate proteins. In the first step, an intermediate thioredoxin-protein complex is formed, which is broken in the second step, releasing the substrate protein in its reduced state. In other words, the disulfide is being transferred from the substrate protein to thioredoxin, or the electrons coming from thioredoxin are shuttled to the protein substrate. The exact reaction mechanism, i.e., the detailed succession of steps in which the reaction takes place, of how this mixed disulfide is broken is not known and has been debated over the last twenty years. With a multidisciplinary approach, combining computational and experimental work, we provide fresh insights into how conformational changes activate the catalytic cysteines with which this universal reduction mechanism is completed with the correct regioselectivity. This work illustrates the strengths of computational approaches in probing phenomena which are otherwise very difficult to investigate experimentally.

## Introduction

Thioredoxin (Trx) is a powerful and universal protein disulfide bond oxido-reductase with a very low redox potential [1]–[3]. All thioredoxins have a similar three-dimensional fold comprising a central core of four β-strands surrounded by three α-helices [4]. All feature a conserved active-site loop containing two redox-active cysteine residues in the sequence Trp-Cys-Gly-Pro-Cys [5] numbered as Trp28 to Cys32 in both *Bacillus subtilis* (Bs_Trx) and *S. aureus* (Sa_Trx) Trx. This numbering will be used throughout. The active site is at the end of an alpha helix, the α1-helix, extending from Lys33 to Glu45. The pKa of the N-terminal active-site cysteine [6]–[9] is significantly lower than the pKa of a cysteine in the absence of a structured protein environment. Under physiological conditions, this low pKa enables thioredoxin to act as a nucleophile, attacking disulfides in proteins [1],[8],[10].

**Figure 1:**
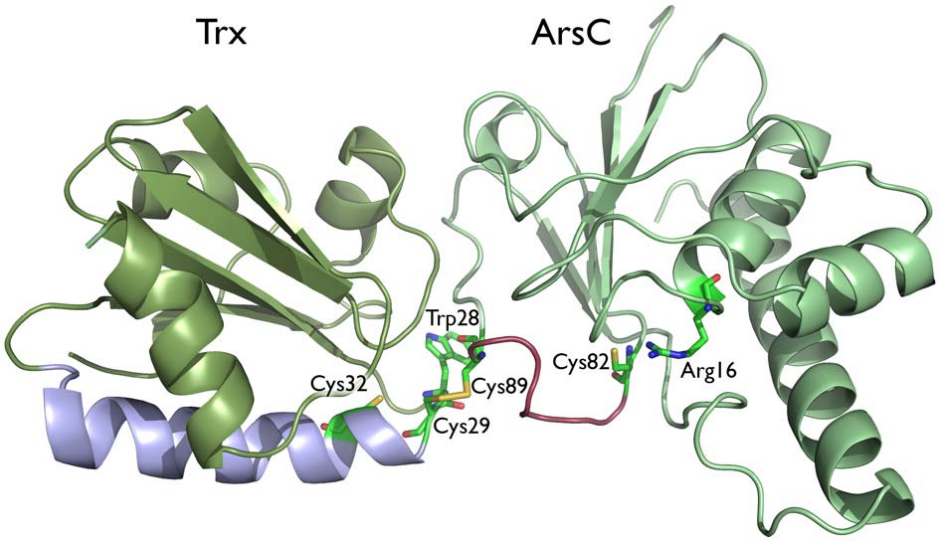
In the structure of the Trx-ArsC mixed disulfide complex the functionally key disulfide between Cys29^Trx^ and Cys89^ArsC^ is formed. The Bs_Trx-ArsC complex (2IPA) with the side chains of residues Cys29^Trx^, Cys32^Trx^, Trp28^Trx^, Arg16^ArsC^, Cys82^ArsC^ and Cys89^ArsC^ in stick representation is shown. The Trx α1-helix is shown in blue; the ArsC looped-out redox helix between Cys82^ArsC^ and Cys89^ArsC^ in pink.

**Figure 2:**
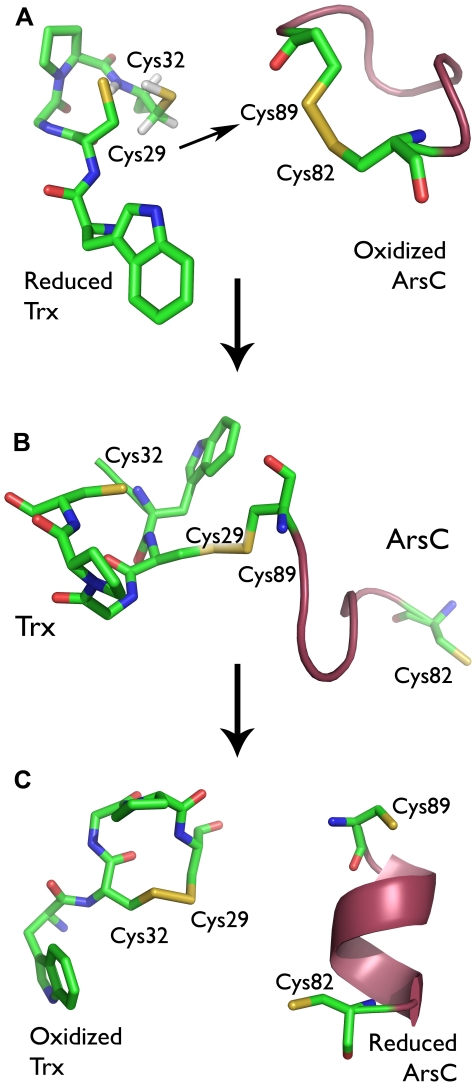
Bs_Trx reduces Bs_ArsC via an intermediate Trx-ArsC complex. **A** Cys29^Trx^ of reduced Bs_Trx (2GZY) nucleophilically attacks Cys89^ArsC^ of the Cys82^ArsC^-Cys89^ArsC^ disulfide of oxidized Bs_ArsC (1Z2E), leading to the formation of the mixed Cys29^Trx^-Cys89^ArsC^ disulfide (2IPA). (**B and C**) Cys32^Trx^ performs a nucleophilic attack on Cys29^Trx^, leading to the release of reduced Bs_ArsC (1Z2D) and oxidized Bs_Trx (2GZZ).

Well-documented endogenous substrates of Trx are the arsenate reductases (ArsC) from the thioredoxin-coupled ArsC family, including *B. subtilis* (Bs_ArsC) and *S. aureus* (Sa_ArsC) arsenate reductase [11]–[14]. ArsC catalyzes the reduction of arsenate [As(V)] to arsenite [As(III)] and is a key enzyme involved in arsenic detoxification [15]. For the reduction of arsenate, Bs_ArsC and Sa_ArsC combine a phosphatase-like nucleophilic displacement reaction in the active site with a distinct intra-molecular disulfide-bond cascade [13], [16]–[18]. Three redox active cysteines are involved (Cys10, Cys82 and Cys89) [19]. After a single catalytic arsenate reduction event, oxidized ArsC exposes a disulfide between Cys82 and Cys89 on a looped-out redox helix [17],[18]. Thioredoxin converts oxidized ArsC back to its initial reduced state [1]. Cys29^Trx^ nucleophilically attacks Cys89^ArsC^ of the Cys82^ArsC^-Cys89^ArsC^ disulfide, leading to the reduction of Cys82^ArsC^ and the formation of the Trx-ArsC mixed disulfide intermediate complex between Cys29^Trx^ and Cys89^ArsC^ (Figure 1 and Figure 2A) [1],[20]. In this complex, Cys32^Trx^ performs a nucleophilic attack on Cys29^Trx^ of the Cys29^Trx^-Cys89^ArsC^ disulfide (Figure 2B). Accordingly, the Trx-ArsC complex dissociates, releasing reduced ArsC and oxidized Trx (Figure 2C).

The mechanism behind the experimentally observed regioselectivity (attack on Cys89^ArsC^ instead of on Cys82^ArsC^ during complex formation and on Cys29^Trx^ instead of on Cys89^ArsC^ during complex dissociation) is not known. Experimentally, it has been observed that Cys32^Trx^ had performed a nucleophilic attack on Cys29^Trx^, but why and how this can occur was not known. A priori, there is another possibility, i.e. a nucleophilic attack by Cys82^Trx^ leading to an unproductive cycle. The regioselectivity analysis gives explanatory insights in the observed mechanism, which could not be understood on the basis of prior information including the experimental structure of the Trx-ArsC complex. This regioselectivity is critical for the function of the involved proteins, and is a reoccurring question within thiol-oxidoreductases. Also, it is not known how Cys32^Trx^ is activated as a thiolate in the Trx-ArsC mixed disulfide complex, resulting in a productive cycle. Especially the deprotonation mechanism of the Cys32^Trx^ thiol is extensively debated [21]–[26].

Although structures of Trx-peptide mixed disulfide complexes have been described, i.e. Trx-Ref-1 [27] and Trx-NFκβ [28], today, the only structure of a Trx-protein mixed disulfide complex is that recently obtained between Trx and Bs_ ArsC [20]. This presents an opportunity to study Trx mixed disulfide complex formation and dissociation. Here, molecular dynamics (MD), pKa calculations, reactivity analysis with conceptual density functional theory (DFT) and biochemical complex formation experiments with Cys-mutants are applied to probe different aspects of the reaction mechanisms of interest: differential reactivity of the cysteines, regioselectivity of the nucleophilic attacks, structure of the Trx-ArsC activated complex, and the role of specific residues such as Asp23^Trx^. These various elements are combined to propose a new and consistent view of the mechanisms, firmly grounded in structural information.

A computational approach is well suited here in particular because it allows the direct investigation of the wild-type sequence with Cys32^Trx^. Also, computation offers the possibility to probe directly the structure, dynamics and energetics of the reacting species in atomic details. That is why computational approaches have made profound contributions to the study of enzymatic reaction mechanisms in recent years, typically combining MD simulations and quantum-mechanical calculations [29].

Nowadays, molecular dynamics (MD) simulations have become an important tool to explore the structural dynamics of proteins and their implications for function [30]. MD simulations have been applied to a number of thiol disulfide oxidoreductases in the Trx superfamilly to determine the structural factors that control the pKa of the thiols [31]–[33]. These studies and others [34]–[37] showed that hydrogen-bonding to cysteine sulphur atoms is crucial to stabilize the thiolate form and influence the reactivity. We have exploited MD simulations in explicit solvent to uncover conformational changes at the Trx-ArsC interface, which provide new insights in the structural factors underpinning the chemistry of the Trx-ArsC complex dissociation.

The NMR structure of the mixed disulfide was used as a *starting point* for MD simulations, pKa and reactivity calculations. The conformational changes at the Trx-ArsC interface during the MD simulations revealed how Cys32^Trx^ can move close enough to Cys29^Trx^ to bring the two sulphur atoms in contact, primed for reaction. This local conformational change is a functionally new conformation, not present in the NMR structure and clearly different from the conformers in the NMR structure of the Trx-ArsC mixed disulfide. In this conformation, the sulphur of Cys32^Trx^ can form two hydrogen-bonds stabilizing its thiolate form. In the following we refer to this conformation as the “activated complex”.

Knowledge of the protonation state of the residues involved in the reaction mechanism is highly important, but the respective pKa values are not always easy to determine experimentally [38]. Therefore, much effort is devoted to develop methods for theoretical pKa computation [38],[39]. Several approaches exist, including classical electrostatics in the context of the Poisson-Boltzmann formalism [36],[40] and more empirically trained models [34]. In the present work, the linear correlation between the natural population analysis (NPA) charge [41] calculated on the sulphur atom of thiols and their experimentally determined pKa [37],[42] is used to quantitatively calculate the cysteine pKa's in thioredoxin, arsenate reductase, and their mixed disulfide complex, including the activated complex. The more negative the NPA charge on the sulphur atom, the higher the tendency to bind a proton, and the higher the thiol pKa is. Calculation of the NPA charge with DFT means that the electronic environment of the sulphur atom is treated explicitly. To account for solvent effects, a continuum solvent model is applied. As such, the NPA-pKa correlation method is better rooted in first principles than the more empirical approaches [34]. The NPA-pKa method was successfully used in the study of the activation of the Cys82 and the Cys89 thiolates in ArsC [42] and in the study of the origin of the pKa perturbation of N-terminal cysteines in α- and 3_10_-helices [37]. The NPA-pKa method remains tractable for proteins, because models including the relevant protein environment for the considered cysteine were designed.

Another aspect of reaction mechanisms related to structure is their regioselectivity. The regioselectivity of the disulfide exchange reactions during Trx-ArsC mixed disulfide complex formation and dissociation can be addressed by the soft acids an bases principle (HSAB) [43],[44]. This principle is defined in the conceptual DFT [45] context and states that hard acids prefer to react with hard bases whereas soft acids prefer to interact with soft bases. Disulfide exchange reactions are soft-soft interactions; as such the softness is used as a reactivity descriptor. The smaller the difference in the local softness (*s*) between the sulphur atoms the more preferred the reactivity between the attacking nucleophilic cysteine (Cys29^Trx^, Cys32^Trx^ and Cys82^ArsC^) and the accepting electrophilic disulfide (Cys82^ArsC^-Cys89^ArsC^ and Cys29^Trx^-Cys89^ArsC^). This might result in several possible reaction paths. Similar reaction paths do not cross according to Klopman's rule [46], and as such the relative energies of the reagents at the ground state (at the beginning of the reaction) correlate with the relative energies at the transition state. An advantage of this conceptual framework is that no activation energies need to be calculated to predict the reaction path. Only reactivities on structures at the beginning of the reaction were calculated. When investigating complex formation, the reactivity analysis was performed on free Trx and ArsC. When addressing complex dissociation, conformers of the Trx-ArsC complex were used. In keeping with the conceptual framework, snapshots of MD simulations representing activated complexes along the reaction path were not used in reactivity analysis.

The local softness (*s*) results from the multiplication of the global softness (*S*) with the Fukui function (*f*) [47]. S is a global property and correlates with the system polarizability [48]. S is the inverse of the chemical hardness, which is the second derivative of the energy with respect to the numbers of electrons, evaluated at a fixed molecular geometry. This derivative can be approximated as the difference between the vertical ionization energy (IE) and the electron affinity (EA). Differentiation of the energy with respect to the external potential *υ*(*r*) (i. e. the potential felt by the electrons due to the nuclei) introduces a local character into the global reactivity descriptors resulting in the Fukui function *f*. *f* is a local descriptor and indicates regions where the molecule preferentially reacts (regioselectivity): i.e. which nucleophilic sulphur atom (*f* ^−^) will attack and which sulphur (*f* ^+^) of the disulfide will receive the electrons. The − and + sign indicate the reactivity towards nucleophilic and electrophlic attacks respectively. *f* ^−^ is related to the electron density of the highest occupied molecular orbital and *f* ^+^ to the density of the lowest unoccupied molecular orbital when electrons are received [49]. The global and local softness and the Fukui function are well established and correlate with reactivity data [45].

In sum, the local softness descriptors are combined with pKa calculations, MD simulations on the Trx-ArsC complex and with biochemical experiments to give fresh insights in the mechanism behind mixed disulfide complex dissociation.

## Results

### pKa's of thiols quantitatively calculated from NPA charges

**Figure 3:**
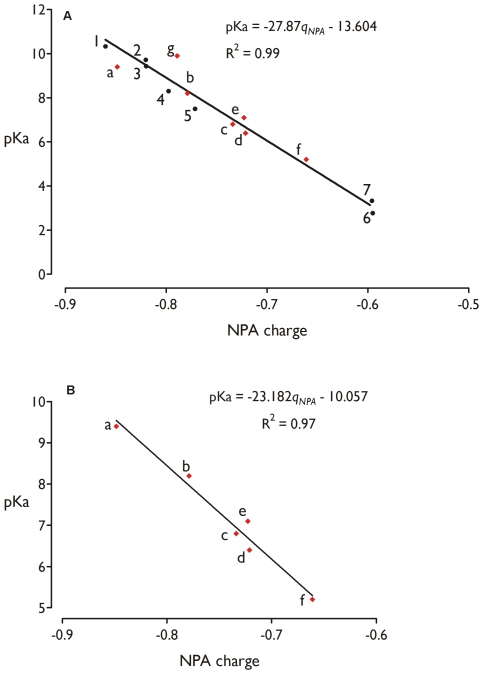
Experimental pKa’s are correlated with the calculated NPA charge. NPA-charge-pKa calibration curve (black circles) obtained **A** for a series of small thiolate molecules (the experimental pKa's are obtained from ref. [74]) and **B** for a series of cysteine residues of Trx and ArsC systems. The data points of the calibration curve B (a–g) are tabulated in Table S1 in Supplemental Data and are also indicated on curve A. trifluoromethanethiol (1), methanethiol (2), mercaptoethanol (3), cysteine (4), trifluoroethanethiol (5), benzenemethanethiol (6) and thioacetic acid (7). Cys89 *S. aureus* ArsC (a), Cys77 *B. subtilis* resA [75],[76] (b), Cys10 *S. aureus* ArsC (c), Cys32 *E. coli* Trx1 [24] (d), Cys29 *S. aureus* P31T C32S Trx1 [9] (e), Cys73 *R. capsulatus* Trx2 [77] (f) and Cys35 *E. coli* Trx1 [24] (g).

The NPA charge has been shown to be an effective descriptor for the pKa. In a series of small substituted thiolate molecules, a linear relationship was obtained between the NPA-charge of the sulphur atom and the experimental pKa value (Figure 3A) [37]. The more negative the NPA charge on the sulphur atom, the higher the tendency to bind a proton and, the more basic (i. e. higher pKa) the compound is. This linear relationship can be used as a calibration curve to quantify the pKa perturbing effect. Remarkably, this initial NPA-pKa correlation [37] obtained for a series of small molecules was found to be directly transferable to the pKa of the cysteine residues in Trx and ArsC (Figure 3A, Table S1 and Figure S4 in Supplemental Data).

The protein environments are represented by models which include all residues interacting with the considered cysteines. The models of all Trxs include the WCPGC active site and the adjacent α1-helix. ArsC is represented by the redox helix region (Cys82-Cys89), Arg16 and Thr11.

The calculated cysteine pKa values are in agreement with the experimentally obtained pKa's with a maximum deviation of 0.5 pKa units (Figure 3A, red diamonds). This confirms that the model systems used for the calculations are appropriate to represent the cysteines in their protein environment. So far, the only outliner is the non-nucleophilic cysteine of *E. coli* Trx1 (Ec_Trx1; data point ‘*g*’ in Figure 3A), which has a severely underestimated pKa. This is likely due to the presence of the nucleophilic cysteine thiolate at 4.7 Å (sulphur-sulphur distance). In the Ec_Trx1 model system used for pKa analysis of this non-nucleophilic cysteine, the negative charge of the neighbouring thiolates, is equally distributed among the Sγ atoms of both residues. As such, no negative charge can build up on the non-nucleophilic cysteine, leading to the underestimation of its pKa. The theoretical pKa estimation via the NPA charge is not suited for two neighbouring negatively charged thiolates, but that is not a situation which dominates or influences particularly the object of our study.

**Figure 4:**
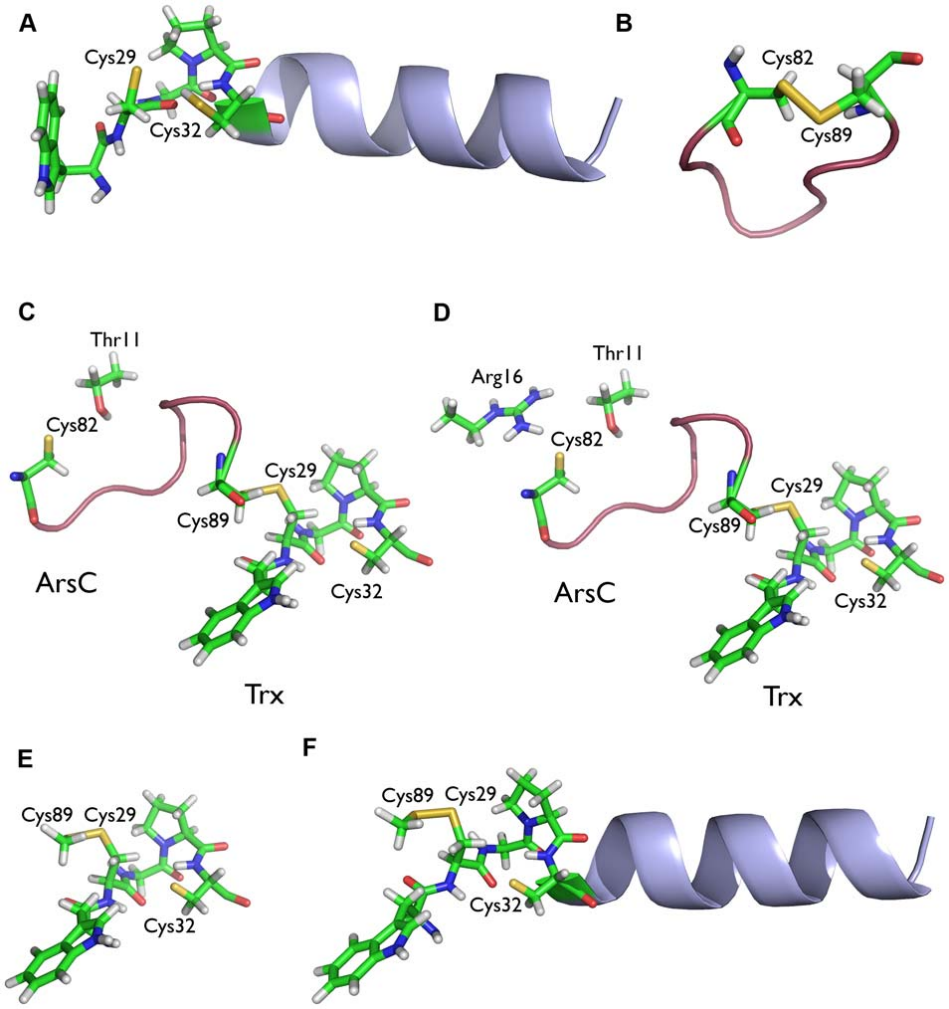
Model systems of Bs_Trx, Bs_ArsC and Bs_Trx-ArsC complex for pKa and reactivity analysis. **A** Reduced Bs_Trx (2GZY) modelled by the Trp28^Trx^-Cys32^Trx^ active site and the Lys33-Glu45 α1-helix (blue). **B** Oxidized Bs_ArsC (1Z2E) represented by the Cys82^ArsC^-Cys89^ArsC^ looped-out redox helix (ArsC_ox). **C** Model of Bs_Trx-ArsC (Trx_ArsC_1), in which no hydrogen bond interactions with Cys32^Trx^ nor with Cys82^ArsC^ are present, represented by the Trp28^Trx^-Cys32^Trx^ Trx active site and the Cys82^ArsC^-Cys89^ArsC^ ArsC redox helix (pink) and Thr11^ArsC^. **D** Model of Bs_Trx_ArsC (Trx-ArsC_2), in which the Cys82^ArsC^S_γ_—Arg16^ArsC^N_η1_ and the Cys32^Trx^S_γ_—Cys29^Trx^N hydrogen bonds are present. The Trp28^Trx^-Cys32^Trx^ Trx active site and the Cys82^ArsC^-Cys89^ArsC^ ArsC redox helix (pink) and Thr11^ArsC^ and Arg16^ArsC^ are included. **E** Trx_ArsC_1_trunc and Trx_ArsC_2_trunc model systems including the Trp28^Trx^-Cys32^Trx^ active site of Trx and Cys89^ArsC^ of the ArsC. **F** Trx_ArsC_2_trunc+helix and Trx_ArsC_1_trunc+helix model systems including everything described under (**E.**) and the Trx Lys33^Trx^-Glu45^Trx^ α1-helix (blue).

The linear correlation between the NPA charge on the sulphur atom and the experimental pKa was adjusted specifically for the data points a–f of the Trx and ArsC systems (Figure 3B, Table S1 in Supplemental Data). This correlation is particularly suited for the active site thiols of the enzymes Trx and ArsC and is used throughout this work to quantitatively calculate the pKa's of the cysteine residues involved in Trx-ArsC complex formation and dissociation. Details on the models used for pKa and reactivity analysis (*vide infra*) of the cysteine residues involved in Trx-ArsC complex formation and dissociation are given in Figure 4.

### Cys29 of Trx nucleophilically attacks Cys89 of oxidized ArsC

With oxidized Bs_ArsC as a substrate, the Cys29^Trx^ nucleophile of reduced Bs_Trx attacks Cys89^ArsC^ of the Cys82^ArsC^-Cys89^ArsC^ disulfide (Figure 2A). This section addresses the reasons behind the experimentally observed regioselectivity of this nucleophilic attack of Cys29^Trx^ on the Cys82^ArsC^-Cys89^ArsC^ disulfide.

In this initial reaction, Cys29^Trx^ is stabilized as a nucleophilic thiolate, in contrast with Cys32^Trx^. Based on the NPA-pKa correlation (Figure 3B), the pKa's of Cys29^Trx^ and Cys32^Trx^ (in respectively the Trx_red_Cys29 and Trx_red_Cys32 models; Figure 4A) in reduced Bs_Trx (2GZY) are calculated to be 5.5 and 8.2 respectively (Table 1).

**Table 1:** The pKa's of the thiols are quantitatively calculated via their respective NPA charges.

	Model system	PDB	Cysteine residue	pKa
**Bs_Trx**	**Trx_red_Cys29 (** **Figure 4A** **)**	2GZY	Cys29^Trx^	5.5
	**Trx_red_Cys32 (** **Figure 4A** **)**	2GZY	Cys32^Trx^	8.2
**Bs_ArsC**	**ArsC_red**	1Z2E	Cys82^ArsC^	6.0
**Bs_Trx-ArsC**	**Trx_ArsC_1_Cys32 (** **Figure 4C** **)**	2IPA	Cys32^Trx^	8.9
	**Trx_ArsC_1_Cys82 (** **Figure 4C** **)**	2IPA	Cys82^ArsC^	7.9
	**Trx_ArsC_1_trunc (** **Figure 4E** **)**	2IPA	Cys32^Trx^	7.9
	**Trx_ArsC_1_trunc+helix (** **Figure 4F** **)**	2IPA	Cys32^Trx^	7.5
	**Trx_ArsC_2_Cys32 (** **Figure 4D** **)**	2IPA	Cys32^Trx^	8.3
	**Trx_ArsC_2_Cys82 (** **Figure 4D** **)**	2IPA	Cys82^ArsC^	6.3
	**Trx_ArsC_2 R16A (** **Figure 4D** **)**	2IPA	Cys82^ArsC^	8.8
	**Trx_ArsC_2_trunc (** **Figure 4E** **)**	2IPA	Cys32^Trx^	8.0
	**Trx_ArsC_2_trunc+helix (** **Figure 4F** **)**	2IPA	Cys32^Trx^	7.2
	**MD snapshot after a simulation time of 14.5 ns in a model similar to Trx_ArsC_2_Cys32**	2IPA	Cys32^Trx^	7.7

Calculated pKa's of the nucleophilic cysteines in Bs_Trx, Bs_ArsC and the Bs_Trx-ArsC complex. The pKa values are obtained via the NPA-pKa correlation presented in Figure 3B.

The minimal difference in the local softness between the sulphur atoms of electrophile and nucleophile favors the nucleophilic attack of Cys29^Trx^ on Cys89^ArsC^ (Table 2). In the Cys82^ArsC^-Cys89^ArsC^ disulfide of oxidized ArsC (1Z2D, Bs_ox in Figure 4B), the Fukui function value of Cys89^ArsC^ is clearly higher than that of Cys82^ArsC^ (Table 3). As such, Cys89^ArsC^ is intrinsically more susceptible to a nucleophilic attack than Cys82^ArsC^. This regioselectivity is largely determined by the side chains of the looped-out redox helix in which the Cys82^ArsC^-Cys89^ArsC^ disulfide is embedded. Indeed, removing the side chains of the redox helix (‘Bs_ox without side chains’ model system) blurs this regioselectivity (Table 3).

**Table 2:** Reactivity analysis of the Bs_Trx-ArsC complex formation: local softness matching.

Δs	Cys29^Trx^/Cys82^ArsC^	Cys29^Trx^/Cys89^ArsC^	Cys32^Trx^/Cys82^ArsC^	Cys32^Trx^/Cys89^ArsC^
**Trx_red – ArsC_ox**	4.73	4.58	6.82	6.66
**Trx_red - ArsC_ox without side chains**	4.52	4.48	6.61	6.57

Reactivity of Cys29^Trx^ and Cys32^Trx^ of Bs_Trx towards the Cys82^ArsC^-Cys89^ArsC^ disulfide of Bs_ArsC as measured by the difference in local softness (Δs).

**Table 3:** Reactivity analysis of the Bs_Trx-ArsC complex formation: softness and Fukui function.

	Model system	Cysteine residue	*S*	*f* ^+^/*f* ^−^	*s* ^+^/*s* ^−^
**Bs_Trx (2GZY)**	**Trx_red_Cys29 (** **Figure 4A** **)**	**Cys29^Trx^**	6.19	0.866	5.36
	**Trx_red_Cys32 (** **Figure 4A** **)**	**Cys32^Trx^**	8.68	0.858	7.45
**Bs_ArsC (1Z2E)**	**ArsC_ox (** **Figure 4B** **)**	**Cys82^ArsC^**	5.17	0.122	0.63
		**Cys89^ArsC^**	5.17	0.152	0.78
**Bs_ArsC (1Z2E)**	**ArsC_ox without side chains (** **Figure 4B** **)**	**Cys82^ArsC^**	5.17	0.162	0.84
		**Cys89^ArsC^**	5.17	0.171	0.88

Global softness (*S*), Fukui function (*f* ^+^ or *f* ^−^) and local softness (*s* ^+^ or *s* ^−^) values of the nucleophilic cysteines in Bs_ArsC and Bs_Trx.

In reduced Bs_Trx (2GZY, Trx_red_Cys29 and Trx_red_Cys32 in Figure 4A), Cys29^Trx^ and Cys32^Trx^ have comparable Fukui function values, corresponding to the same intrinsic reactivity. However, here, Cys29^Trx^ has a lower softness than Cys32^Trx^, explaining (in addition to the pKa values) its higher reactivity towards the less soft Cys82^ArsC^-Cys89^ArsC^ disulfide (Table 3). So, pKa calculations combined with reactivity analysis explain the regioselectivity of the first reaction in the catalytic cycle leading to reduction of ArsC by Trx.

### Hydrogen bonds stabilize the thiolate form of Cys32^Trx^ and of Cys82^ArsC^ in the mixed disulfide complex

The NMR structure of the Bs_Trx-ArsC complex (PDB code 2IPA) [1] and the derived MD simulations (75 ns) have been used to investigate the dynamics of the hydrogen bond interactions formed with the sulphur atom of Cys32^Trx^ and of Cys82^ArsC^. Indeed, hydrogen bonds to the sulphur can influence its reactivity by stabilizing its thiolate form (see Introduction).

The NMR structure of the Trx-ArsC mixed disulfide was obtained after mutation of Cys32 and Cys82 to serines. These serines were converted to the wild-type cysteines by straightforward modeling in the 21 conformers of the NMR structure. In seven out of the 21 conformers, Cys82^ArsC^ forms a hydrogen bond with Arg16^ArsC^, and in eight of them Cys82^ArsC^, forms a hydrogen bond with Thr11^ArsC^. Cys32^Trx^ is hydrogen-bonded to the N-atom of the Cys29^Trx^ backbone (Figure 4D) in eleven conformers. In eight conformers, no hydrogen bonds are formed with Cys82^ArsC^ or with Cys32^Trx^ (Figure 4C). This simple analysis suggested that both protonation states of Cys82^ArsC^, neutral and thiolate, should be considered, since in the 21 conformers, Cys82^ArsC^ forms either no or two hydrogen bonds.

**Figure 5:**
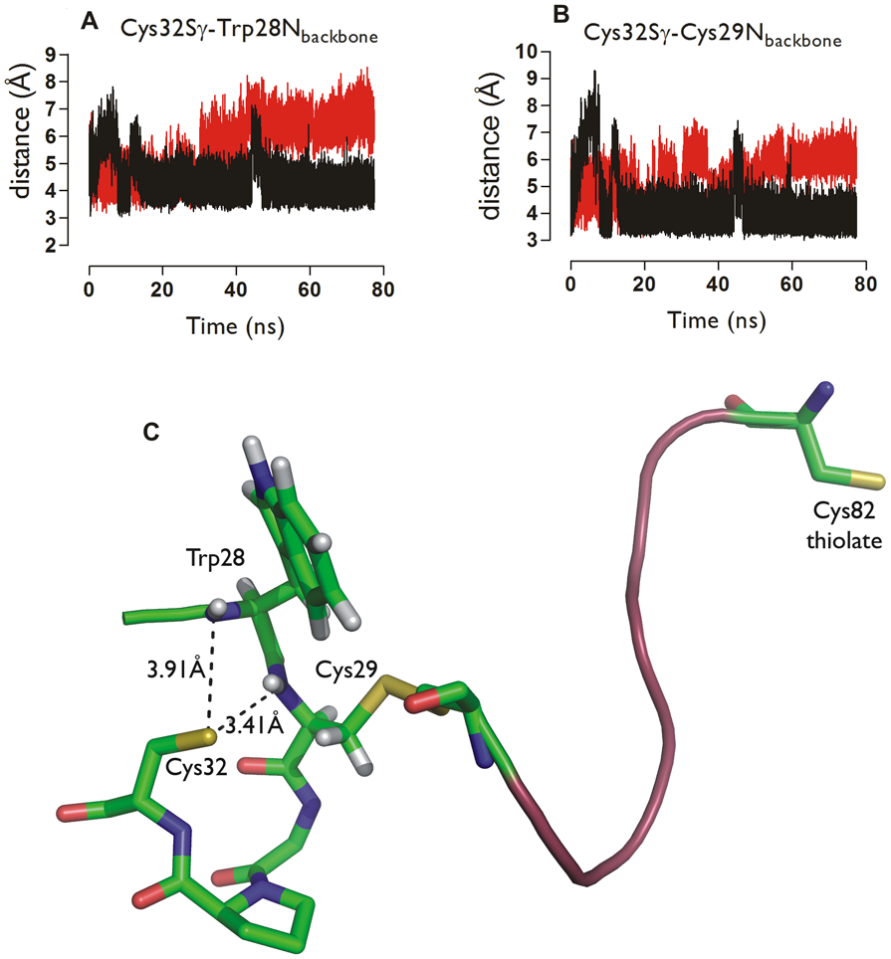
Hydrogen bonding of Cys32^Trx^ in the mixed disulfide of Bs_Trx-ArsC. Time course in the MD simulation of the distance between **A** Cys32^Trx^S_γ_ and Trp28^Trx^N and between **B** Cys32^Trx^S_γ_ and Cys29^Trx^N, with ionized (black) and neutral (red) Cys82^ArsC^. **C** Example of one MD snapshot with the Cys32^Trx^S_γ_—Cys29^Trx^N and Cys32^Trx^S_γ_—Trp28^Trx^N hydrogen bonds introduced, when Cys82^ArsC^ is deprotonated.

Starting from a conformer of the NMR Trx-ArsC structure devoid of hydrogen bonds to Cys32^Trx^ and Cys82^ArsC^, two MD simulations were set up, with Cys82^ArsC^ modeled as neutral or as a thiolate. When Cys82^ArsC^ is deprotonated, the following hydrogen bonds are formed during the simulation (Figure 5 and Figure S1 in Supplemental Data): Cys82^ArsC^S_γ_—Arg16^ArsC^N_η1_ (15% of the time), Cys82^ArsC^S_γ_—Arg16^ArsC^N_ε_ (1% of the time), Cys82^ArsC^S_γ_—Thr11^ArsC^N (94% of the time), Cys82^ArsC^S_γ_—Thr11^ArsC^O_γ_ (74% of the time), Cys82^ArsC^S_γ_—Arg108^ArsC^N_η2_ (48% of the time), Cys82^ArsC^S_γ_—Arg108^ArsC^N_η1_ (40% of the time), Cys32^Trx^S_γ_—Cys29^Trx^N (60% of the time) and Cys32^Trx^S_γ_—Trp28^Trx^N (23% of the time). Cys32^Trx^ is simultaneously hydrogen bonded to Cys29^Trx^N and Trp28^Trx^N 20% of the time. Not surprisingly, hydrogen bonds to Cys82^ArsC^ were formed significantly less frequently in the MD simulation where it is neutral (Cys82^ArsC^S_γ_—Arg16^ArsC^N_η1_ (19% of the time), Cys82^ArsC^S_γ_—Arg16^ArsC^N_ε_ (1% of the time), Cys82^ArsC^S_γ_—Thr11^ArsC^N (14% of the time), Cys82^ArsC^S_γ_—Thr11^ArsC^O_γ_ (1% of the time), Cys82^ArsC^S_γ_—Arg108^ArsC^N_η2_ (0% of the time), Cys82^ArsC^S_γ_—Arg108^ArsC^N_η1_ (0% of the time). In addition, Cys32^Trx^ was hydrogen bonded to Cys29^Trx^N and to Trp28^Trx^N for respectively 1.6% and 0.5% of the time, much less frequently than when Cys82^ArsC^ is treated as thiolate. This is a strong indication that the protonation state of Cys82^ArsC^ influences the behavior of Cys32^Trx^. Of course, the exact numbers presenting the statistics from the simulations, e.g. regarding how frequently a hydrogen-bond is formed, are subject to the length of a simulation and its particular starting point, and are thus expected to vary without changing the main trends.

The formed hydrogen bonds influence the pKa of both Cys32^Trx^ and Cys82^ArsC^. In the Bs_Trx-ArsC complex, both the Cys32^Trx^S_γ_—Cys29^Trx^N hydrogen bond and the α1-helix of Trx stabilize Cys32^Trx^ as a thiolate. When Cys32^Trx^ is not hydrogen bonded, its calculated pKa is 8.9 (Trx_ArsC_1_Cys32, Figure 4C) (Table 1). In the presence of the Cys32^Trx^S_γ_—Cys29^Trx^N hydrogen bond (Trx_ArsC_2_Cys32, Figure 4D), the pKa drops to 8.3.

To account for the effect of the α1-helix, we designed the Trx_ArsC_1_trunc and Trx_ArsC_2_trunc (Figure 4E–F), in which only Cys89^ArsC^ of the ArsC part is included. Here, the α1-helix additionally decreases the pKa of Cys32^Trx^ with 0.4 and 0.8 pKa units respectively.

Cys82^ArsC^ has a pKa of 8.1 (Table 1) in the absence of hydrogen bond interactions (Trx_ArsC_1_Cys82, Figure 4C). Its pKa drops to 6.3 when Arg16^ArsC^ comes within hydrogen bonding distance (Trx_ArsC_2_Cys82, Figure 4D).

In sum, in the Bs_Trx-ArsC complex, hydrogen bonds formed with the sulphurs of both Cys32^Trx^ and Cys82^ArsC^ are functionally important and stabilize the thiolate form of these cysteines.

### Cys32^Trx^ nucleophilically attacks Cys29^Trx^ in the Bs_Trx-ArsC mixed disulfide complex

In the Bs_Trx-ArsC complex, dissociation takes place via the nucleophilic attack of Cys32^Trx^ on Cys29^Trx^ of the mixed Cys29^Trx^-Cys89^ArsC^ disulfide (Figure 2B). Given that both Cys82^ArsC^ and Cys32^Trx^ can be stabilized as a thiolate, both are considered as possible nucleophiles.

The nucleophilic attack of Cys32^Trx^ on Cys29^Trx^ is the favored reaction. Comparing the differences in local softness between Cys32^Trx^/Cys82^ArsC^ (potentially attacking nucleophiles) and Cys29^Trx^/Cys89^ArsC^ (potentially attacked electrophiles) shows a minimal local softness difference between Cys32^Trx^ and Cys29^Trx^ (Table 4). The Fukui function values of Cys29^Trx^-Cys89^ArsC^ obtained when Cys32^Trx^ is deprotonated (Trx_ArsC_2_Cys32, Figure 4D) are higher than those obtained when Cys32^Trx^ is protonated (Trx_ArsC_2_Cys82, Table 4). Thus, the deprotonation of Cys32^Trx^ in the Trx_ArsC_2_Cys32 model increases the susceptibility of the Cys29^Trx^-Cys89^ArsC^ disulfide to a nucleophilic attack. This is consistent with the need for an activated nucleophile in order to perform the reaction. When Cys32^Trx^ is deprotonated, Cys29^Trx^ is the preferred target for nucleophilic attack (Figure 2, Table 5), since the Fukui function of Cys29^Trx^ in the mixed disulfide is clearly higher than that of Cys89^ArsC^. The ArsC redox helix is essential for the regioselectivity, since in the absence of this redox helix (Trx_ArsC_2_trunc), the difference in the Fukui function values between Cys29^Trx^ and Cys89^ArsC^ is fading. The Trx α1-helix (Trx_ArsC_2_trunc+helix) is not essential for the regioselectivity since the Fukui function values are not affected by adding this helix to the model system (Table 5).

**Table 4:** Reactivity analysis of the Bs_Trx-ArsC complex dissociation: softness matching.

Δs	Cys32^Trx^/Cys29^Trx^	Cys32^Trx^/Cys89^ArsC^	Cys82^ArsC^/Cys29^Trx^	Cys82^ArsC^/Cys89^ArsC^
**Trx_ArsC_2_Cys32**	2.48	4.86	4.21	6.60
**Trx_ArsC_2_Cys82**	/	/	6.21	6.86
**Trx_ArsC_2_Cys82 Arg16**	/	/	6.17	6.52
**Trx_ArsC_2_trunc**	3.40	4.00	/	/
**Trx_ArsC_2_trunc+helix**	3.11	4.52	/	/

Reactivity of Cys32^Trx^ and Cys82^ArsC^ towards the Cys29^Trx^-Cys89^ArsC^ mixed disulfide as measured by the difference in local softness (Δs).

/: not determined.

**Table 5:** Reactivity analysis of the Bs_Trx-ArsC complex dissociation: softness and Fukui function.

Bs_Trx-ArsC complex (2IPA)	Cysteine residue	*S*	*f* ^+^/*f* ^−^	*s* ^+^/*s* ^−^
**Trx_ArsC_2_Cys32 (** **Figure 4D** **)**	**Cys32^Trx^**	6.47	0.865	5.59
	**Cys29^Trx^**	5.97	0.522	3.12
	**Cys89^ArsC^**	5.97	0.122	0.73
	**Cys82^ArsC^**	8.59	0.856	7.33
**Trx_ArsC_2_Cys82 (** **Figure 4D** **)**	**Cys82^ArsC^**	8.57	0.856	7.34
	**Cys29^Trx^**	5.59	0.203	1.14
	**Cys89^ArsC^**	5.59	0.087	0.49
**Trx_ArsC_2_Cys82 Arg16A (** **Figure 4D** **)**	**Cys82^ArsC^**	8.04	0.863	6.64
	**Cys29^Trx^**	6.21	0.124	0.77
	**Cys89^ArsC^**	6.21	0.067	0.42
**Trx_ArsC_2_trunc (** **Figure 4E** **)**	**Cys32^Trx^**	6.47	0.865	5.60
	**Cys29^Trx^**	5.14	0.428	2.20
	**Cys89^ArsC^**	5.14	0.312	1.61
**Trx_ArsC_2_trunc+helix (** **Figure 4F** **)**	**Cys32^Trx^**	6.37	0.865	5.51
	**Cys29^Trx^**	5.21	0.459	2.39
	**Cys89^ArsC^**	5.21	0.189	0.98

Global softness (*S*), Fukui function (*f* ^+^ or *f* ^−^) and local softness (*s* ^+^ or *s−*) values of the nucleophilic cysteines in the Bs_Trx-ArsC complex.

In the Trx_ArsC_2_Cys32 model (Figure 4D), Cys32^Trx^ has only a slightly higher Fukui function value than Cys82^ArsC^. The higher reactivity of Cys32^Trx^ compared to Cys82^ArsC^ towards the less soft Cys29^Trx^-Cys89^ArsC^ disulfide is consistent with the lower softness of Cys32^Trx^ compared to Cys82^Trx^ (Table 5).

This regioselectivity analysis was repeated with the Trx_ArsC_1 model (Figure 4C), confirming the obtained results (results not shown), indicating that our conclusions are not particularly sensitive on structural models, if they include enough relevant protein environment.

**Figure 6:**
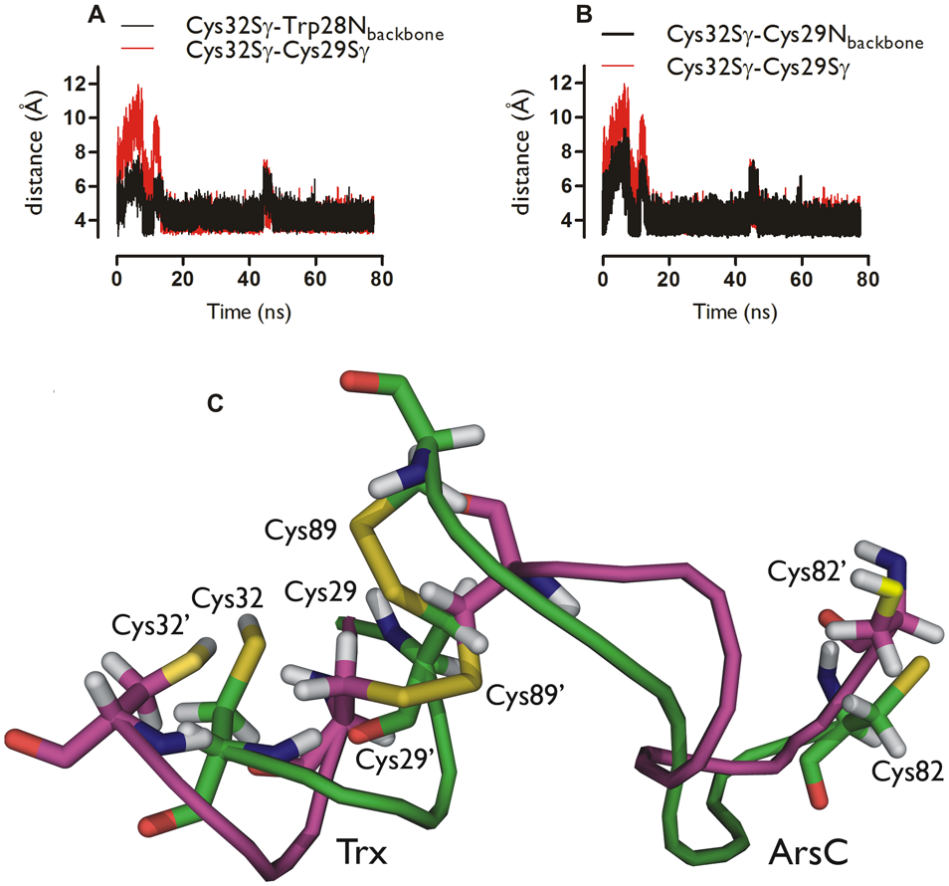
A conformational change brings Cys32^Trx^ in contact with Cys29^Trx^. Time course in the MD simulation (ionized Cys82^ArsC^) of the distance between Cys29^Trx^S_γ_ and Cys32^Trx^S_γ_ (red) and Cys32^Trx^S_γ_ and Trp28^Trx^N (**A**, black) and Cys32^Trx^S_γ_ and Cys29^Trx^N (**B**, black). **C** Superposition of the Trx active site (Trx28^Trx^-Cys32^Trx^) and the ArsC redox helix (Cys82^ArsC^-Cys89^ArsC^) of the Bs_Trx-ArsC complex at 0 ns (purple) and 14.5 ns (green) simulation time showing the conformational change associated with the approach of Cys32^Trx^ to Cys29^Trx^ during the MD simulation (ionized Cys82^ArsC^).

In addition, geometric factors are at work. During both MD simulations of the Trx-ArsC complex, Cys82^ArsC^ (and its Ser82^ArsC^ equivalent in the NMR structure with PDB code 2IPA) is more than 15 Å away from Cys29^Trx^, while a local conformational change brings Cys32^Trx^ up to 3.7 Å of Cys29^Trx^ in the simulation with ionized Cys82^ArsC^ (Figure 6A). In this simulation, the sulphur atoms of Cys32^Trx^ and Cys29^Trx^ are within 4.0 Å of each other 50.7% of the time. We refer to this new conformation where Cys32^Trx^ comes in contact with Cys29^Trx^ (not present in the PDB structure 2IPA) as the activated complex. Indeed, a geometric proximity between sulphurs is a pre-requisite to a reaction between them. Such proximity was not observed between Cys29^Trx^ and Cys82^ArsC^ in the MD simulations.

**Figure 7:**
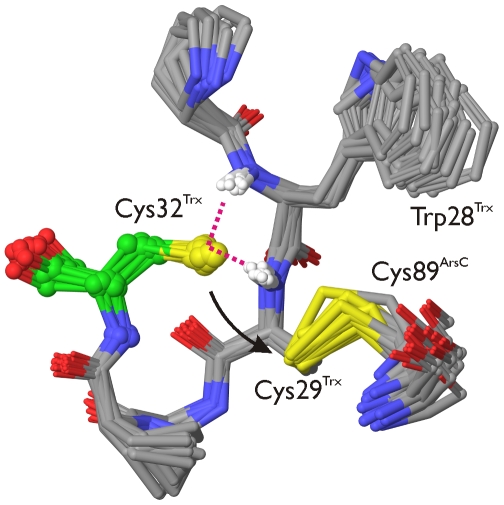
Cys32^Trx^ is primed for nucleophilic attack in the Trx-ArsC activated complex. Selected snapshots from an MD simulation of the Trx-ArsC complex (ionized Cys82^ArsC^), presenting the structural basis of the activation of Cys32^Trx^ (green carbons, ball and sticks) for its nucleophilic attack onto Cys29^Trx^. In these snapshots, Cys32^Trx^S_γ_ is hydrogen-bonded (magenta dotted lines) to the amide NH groups of both Trp28^Trx^ and Cys29^Trx^. Also, the sulphur atoms of Cys32^Trx^ and Cys29^Trx^ are within 4.5 Å of each other. The hydrogen bonds satisfy the geometric criteria: Cys32^Trx^S_γ_—Trp28^Trx^N≤4 Å and Cys32^Trx^S_γ_—Cys29N^Trx^≤4 Å, with the corresponding angles between S_γ_ and the N−H vectors being ≥150 degrees. The hydrogen-bonds donated to the sulphur of Cys32^Trx^ lower its pKa to 7.4 (Table 4), corresponding to a significant population of the thiolate form of Cys32^Trx^. This thiolate being close to Cys29^Trx^, it is in effect primed for nucleophilic attack onto Cys29^Trx^ (black arrow). In combination with the supporting reaction analysis, pKa calculations and complex formation experiments (main text), the conformations and interactions shown here are proposed to underpin the dissociation mechanism of the Tx-ArsC complex.

The conformational change bringing Cys32^Trx^ in contact with Cys29^Trx^ is associated with the formation of the key hydrogen bonds (Figure 6 B–C), stabilizing Cys32^Trx^ as a thiolate, primed for a nucleophilic attack on Cys29^Trx^. The pKa of Cys32^Trx^ was calculated in selected snapshots of the activated complex where both the Cys32^Trx^Sγ—Cys29^Trx^N and the Cys32^Trx^Sγ—Trp28^Trx^N hydrogen bonds are present and particularly strong (based on geometric criteria, see Figure 7). These calculations were performed with models similar to the Trx_ArsC_2_Cys32 model (Table 6). The snapshots of interest were observed at various stages during the simulation, including towards its end, well past what could be considered an equilibration phase. The results indicate that the introduction of the Cys32^Trx^S_γ_—Trp28^Trx^N hydrogen bond allows for an extra pKa decrease to 7.4 compared to the Trx_ArsC_2_Cys32 model, in which only the Cys32^Trx^S_γ_—Cys29^Trx^N hydrogen bond is present. These results indicate that the pKa of Cys32^Trx^ can be instantaneously being low. From a mechanistic point of view, it is enough to deprotonate Cys32^Tx^ for a split second for the subsequent reaction of interest to take place. Indeed, reactivity may not be determined by the average value of the pKa, but may be gated by the occasional low values. Therefore, it is legitimated to select some snapshots of special interest.

**Table 6:** The pKa's of the thiols in the activated complex at different time points of the MD simulation.

Time point in the MD simulation (ns)	Cys32^Trx^ pKa
17.276	8.3
17.943	7.8
30.804	7.8
38.716	7.4
55.687	8.0
60.922	7.8
60.924	7.4
62.487	8.0
63.578	8.2

Calculated pKa's of Cys32^Trx^ in the activated complex in selected snapshots where both the Cys32^Trx^S_γ_—Cys29^Trx^N and the Cys32^Trx^S_γ_—Trp28^Trx^N hydrogen bonds are present and particularly strong (SG…N distance<4 Å and SG…H-N angle>150 deg, with both Trp28-NH and Cys29-NH.) in a model similar to the Trx_ArsC_2_Cys32 model.

### The role of Asp23^Trx^ and of the leaving thiol group in the deprotonation of Cys32^Trx^ revisited

**Figure 8:**
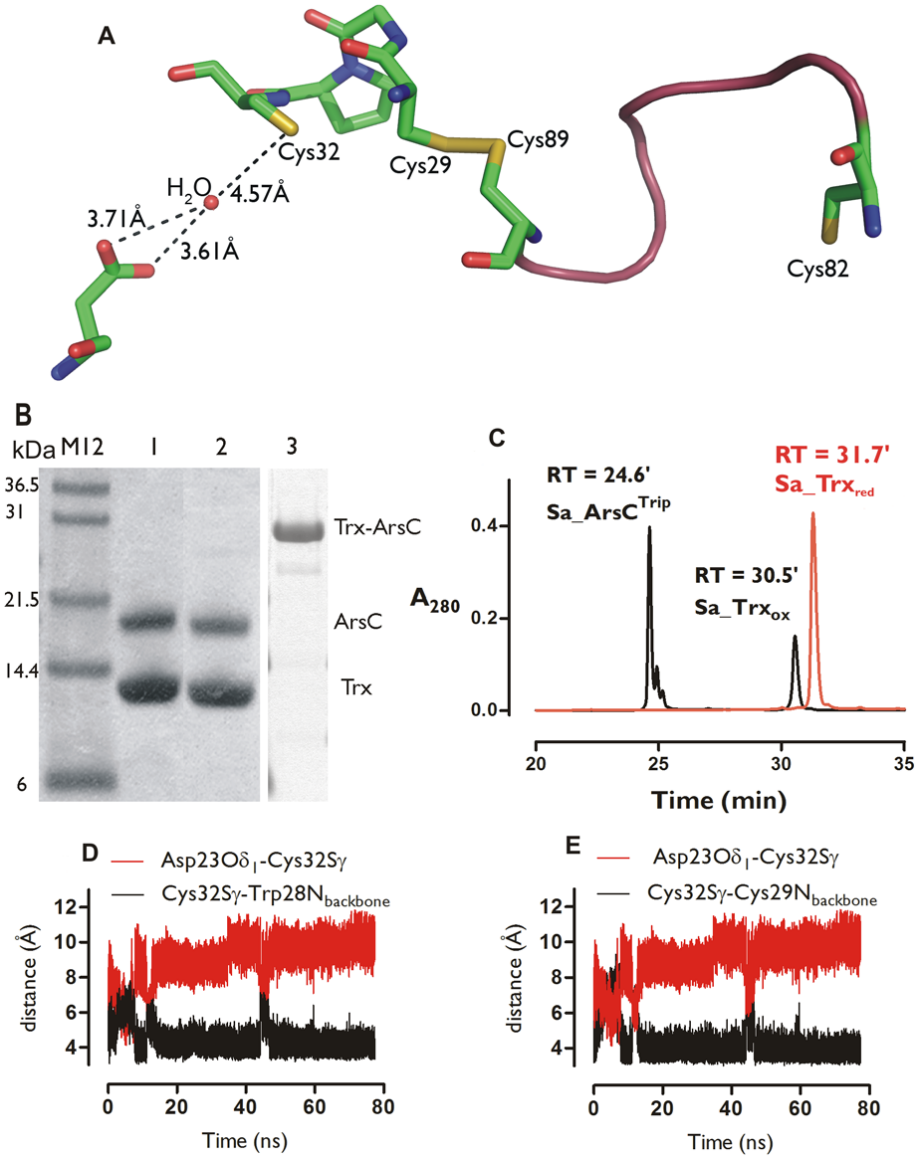
The putative role of Asp23^Trx^ in the deprotonation of Cys32^Trx^ revisited. **A** Water is sometimes proposed to assist the deprotonation of Cys32^Trx^ by Asp23^Trx^. The position of the water molecule in the Bs_Trx-ArsC complex was obtained by modeling. **B** Evaluation of the wild type Sa_Trx-Sa_ArsC^Trip^ and the Sa_Trx D23A-Sa_ArsC^Trip^ complex formation on a non-reducing SDS-PAGE (pH 7.5). No stable complex is detected. (1. Sa_Trx+Sa_ArsC^Trip^; 2. Sa_Trx D23A+Sa_ArsC^Trip^ 3. Positive control band of the Sa_Trx-ArsC complex [1]). (**C.**) Reversed phase-analysis of the reaction products after complex formation between Sa_Trx D23A and Sa_ArsC^Trip^. **E D** Time course in the MD simulation (ionized Cys82^ArsC^) of the distance between Asp23^Trx^O_δ1_ and Cys32^Trx^S_γ_ (red) and Cys32^Trx^S_γ_ and Trp28^Trx^N (**D**, black) and Cys32^Trx^S_γ_ and Cys29^Trx^N (**E**, black).

Although not within hydrogen bonding distance of Cys32^Trx^ (>6 Å), Asp23^Trx^ has been suggested to be the key residue for the activation of Cys32^Trx^ as a nucleophile [26]. Hydrogen bonding via a structurally conserved water molecule located between Asp23^Trx^ and Cys32^Trx^ has been put forward for such activation (Figure 8A) [23],[26]. We revisited the suggested role of Asp23^Trx^ by biochemical experiments and MD simulations.

Complex formation of wild type Sa_Trx with Sa_ArsC C10S/C15A/C82A triple mutant (Sa_ArsC^Trip^) via a 2-nitro-5-mercaptobenzoic acid (TNB)-mixed disulfide with Cys89^ArsCtrip^ (see Experimental procedure section) showed the release of TNB^−^. This means that Cys29^Trx^ attacks Cys89^ArsCtrip^, indicating complex formation (Figure S2 in Supplemental data). Evaluation on non-reducing SDS-PAGE showed two bands, corresponding to ArsC and Trx. No Trx-ArsC complex could be detected (Figure 8B), indicating the dissociation of the mixed disulfide. Reaction with a Trx D23A mutant gave a similar result. With Trx D23A, we observed TNB^−^ release, indicating complex formation, and only the two bands corresponding to Trx and ArsC were detected on a non-reducing SDS-PAGE (Figure 8B). So, in the absence of Asp23^Trx^ as putative nucleophile activator, the Trx-ArsC complex was formed but still able to dissociate. We also evaluated the oxidation state of Sa_Trx D23A on reversed phase chromatography. Before the reaction, Sa_Trx D23A was in its reduced form, while after the reaction Sa_Trx D23A was oxidized (Figure 8C). This proves that Sa_Trx D23A reduces its substrate, consistent with Cys32^Trx^ being activated as a nucleophilic thiolate and this independently of Asp23^Trx^.

The assumed role of Asp23^Trx^ was also investigated by MD experiments on Bs_Trx-ArsC. When Cys82^ArsC^ is ionized, the distance of Asp23O_δ1_ (or Asp23O_δ2_) to Cys32S_γ_ increased to 10 Å and this increase was maintained during the remainder of the simulation up to 75 ns. Associated with this movement, the Cys32^Trx^S_γ_—Cys29^Trx^N and Cys32^Trx^S_γ_—Trp28^Trx^N hydrogen bonds are introduced, forming the activated complex (Figure 8 D, E). This adds to the view that Cys32S_γ_ is activated by hydrogen bonds to the protein backbone rather than interactions with Asp23, consistent with the results from the complex formation experiments.

Recently it was suggested that when the N-terminal thiolate of Trx attacks its substrate disulfide to form a mixed disulfide complex, the leaving thiol group deprotonates the C-terminal active site thiol of Trx [21]. With ArsC as substrate, Cys82^ArsC^ would deprotonate Cys32^Trx^ when Cys29^Trx^ attacks the Cys82^ArsC^-Cys89^ArsC^ disulfide. In this hypothesis, mutating Cys82^ArsC^ to Ala would trap the mixed disulfide complex as a stable entity, which we did not observe. In complexation experiments between wild type Sa_Trx, Sa_Trx D23A and Sa_ArsC^Trip^ in which Cys82^ArsC^ is mutated to alanine, Trx-ArsC complex formation was observed via TNB^−^ release. Neither wild type Sa_Trx nor Sa_Trx D23A formed a stable complex with Sa_ArsC^Trip^ as visualized on non-reducing SDS-PAGE (Figure 7B–C). Our experiments do not support the mechanism proposed in Carvalho *et al* [21],[32],[33],[42],[50].

## Discussion

The intention of this work is to explain the result of the Trx-ArsC mixed disulfide dissociation seen experimentally, since this was not understood on the basis of the experimental structure of the Trx-ArsC complex. The used methods to calculate pKa, regioselectivity and MD are robust and of state-of-the-art quality in the field [31]–[33],[42],[50].

Before forming a mixed disulfide between Trx and ArsC, Cys29^Trx^ has to be stabilized as a thiolate to nucleophilically attack the exposed Cys82^ArsC^-Cys89^ArsC^ disulfide in oxidized ArsC. In the active site of reduced Bs_Trx, both the α1-helix and the Cys29^Trx^S_γ_—Cys32^Trx^NH hydrogen bond stabilize Cys29^Trx^ as a thiolate. In the presence of the Cys29^Trx^S_γ_—Cys32^Trx^NH hydrogen bond the calculated pKa of Cys29^Trx^ (5.5) is remarkably lower than the experimentally determined pKa of the corresponding residue in Ec_Trx1 (7.1) [24] or Sa_Trx (7.1) [1], in which the Cys29^Trx^S_γ_—Cys32^Trx^NH hydrogen bond is not present. The calculated pKa of the C-terminal cysteine of the WCGPC catalytic site (Cys32^Trx^) is 8.1 (Table 1), and only slightly higher in Sa_Trx (pKa∼9)(^24^). The pKa difference between the Trx active site N-terminal and C-terminal cysteines implies that Cys29^Trx^ and not Cys32^Trx^ will attack the Cys82^ArsC^-Cys89^ArsC^ disulfide in oxidized ArsC. These pKa arguments are concordant with the reactivity analysis: being less soft than Cys32^Trx^, Cys29^Trx^ is more reactive towards the less soft Cys82^ArsC^-Cys89^ArsC^ disulfide.

The regioselectivity of Cys29^Trx^ for Cys89^ArsC^ is difficult to rationalize based on the pKa of the attacking nucleophile, or on the negative correlation between the rate of thiol-disulfide exchange reactions and the pKa of the leaving thiol group [51]–[54]. Upon formation of the mixed disulfide complex, either Cys82^ArsC^ or Cys89^ArsC^ can be the leaving thiol (Figure 2). Initially in ArsC, these thiols are flanking a flexible short helix – the so-called redox helix -, but during a single catalytic cycle, this redox helix unrolls, exposing the Cys82^ArsC^-Cys89^ArsC^ disulfide [17]. As the chemical environment of Cys82^ArsC^ and Cys89^ArsC^ changes the moment the helix starts to unfold, their respective pKa values will change. We were not able to determine the pKa values of the leaving Cys82^ArsC^ and Cys89^ArsC^ thiols, neither experimentally nor by calculation since structural information of ArsC with an unfolded redox helix and with both cysteines reduced is lacking. However, we obtained information regarding the selectivity of the nucleophilic attack with a DFT reactivity analysis. The unfolded redox helix in oxidized ArsC sets a higher Fukui function value for Cys89^ArsC^ compared to Cys82^ArsC^ (Table 3). Thus, Cys89^ArsC^ is intrinsically more reactive towards a nucleophilic attack than Cys82^ArsC^, leading to the formation of the Cys29^Trx^-Cys89^ArsC^ mixed disulfide and the release of Cys82^ArsC^.

In a productive cycle, the Cys29^Trx^-Cys89^ArsC^ mixed disulfide is dissociated by the nucleophilic attack of Cys32^Trx^, leading to oxidized Trx and reduced ArsC [13],[16],[17]. The deprotonation mechanism of the Cys32^Trx^ thiol has been debated for more than twenty years [21]–[26]. Structural analyses, kinetic assays, site directed mutagenesis and pKa titrations have led to the suggestion that Asp23^Trx^ may deprotonate Cys32^Trx^ [23]–[26]. Recently it was suggested that the leaving thiol group (here Cys82^Trx^) of the disulfide attacked during the first step deprotonates the C-terminal active site thiol of Trx [21]. Yet, our experiments strongly indicate that the Trx-ArsC mixed disulfide still dissociates when Cys82^ArsC^ is mutated to Ala. In addition, our MD simulations strongly suggest that the local conformational changes around the mixed disulfide (Figure 6C) are responsible for the introduction of extra hydrogen bonds with the Cys32^Trx^ sulphur (Figure 5C).

Experimentally, it is not possible to measure the pKa of Cys32^Trx^ in the Trx-ArsC complex, since the complexes formed between wild type Sa_Trx and Sa_ArsC^Trip^, and between the Sa_Trx D23A mutant and Sa_ArsC^Trip^ were only transient. Due to the transient nature of the Trx-ArsC complex experimental measurements using the wild type sequence is a true challenge. This is in particular the case regarding pKa measurements and probing conformational changes leading to the dissociation. Further, the pKa of Cys32^Trx^ is lowered by hydrogen bonds to the protein backbone. Thus, the role of these hydrogen bonds cannot be probed by additional mutagenesis. Therefore, in the present context, calculations fill a gap on questions that are currently experimentally out of reach, providing a more detailed picture of complex dissociation.

The Cys32^Trx^S_γ_—Cys29^Trx^N hydrogen bond (Trx_ArsC_2 model, Figure 4D), present during 60.0% of the time of the MD simulation, decreases the Cys32^Trx^ pKa from 8.9 to 8.3 (Table 1). This was calculated from the Trx_ArsC_1_Cys32 and the Trx_ArsC_2_Cys32 model respectively. In selected snapshots of the activated complex where both the Cys32^Trx^S_γ_—Cys29^Trx^N and the Cys32^Trx^S_γ_—Trp28^Trx^N hydrogen bonds are present (and particularly strong based on geometric criteria), the pKa of Cys32^Trx^S_γ_ drops to 7.4, sufficient for the dissociation to proceed (Table 6, Figure 7). For the mixed-disulfide complex to dissociate, it is sufficient that Cys32^Trx^ is transiently stabilized as a thiolate in the activated complex. Thus, instantaneous low values of the pKa of Cys32^Trx^ are expected to be more relevant than the corresponding average value. Concomitant with the introduction of these hydrogen bonds, Cys32^Trx^S_γ_ approaches the sulphur of Cys29^Trx^. We refer to this conformation where Cys32^Trx^ is primed for its nucleophilic attack onto Cys29^Trx^S_γ_ as the “activated complex”, as it is functionally relevant, and different from any conformation present in the NMR structure of the complex (PDB code: 2IPA). In sum, the pKa of Cys32^Trx^ clearly depends on the presence of hydrogen bonds. These hydrogen bonds stabilize the thiolate form of Cys32^Trx^.

Additionally, Cys32^Trx^ is part of the α1-helix in Trx accounting for another decrease of the pKa up to 0.8 pKa units. This α1-helix in Trx is solvent exposed and as such the effective dipole moment influencing the pKa of Cys32^Trx^ is relatively low. The effect of the Cys32^Trx^S_γ_—Cys29^Trx^N hydrogen bond and the α1-helix on the calculated pKa is rather small, but can be considered as significant seen the very good correlation between NPA and pKa (Figure 3B).

MD snapshots clearly strongly indicate the activation of Cys32^Trx^ as a nucleophile via a conformational change, which brings Cys32^Trx^ up to 3.7 Å to Cys29^Trx^ (Figure 6A) concurrently with the formation of the Cys32^Trx^S_γ_—Cys29^Trx^N and Cys32^Trx^S_γ_—Trp28^Trx^N hydrogen bonds. These hydrogen-bonds form even though Cys32^Trx^S_γ_ was simulated in its neutral form. Simulating Cys32^Trx^S_γ_ as a thiolate would presumably provide an even stronger driving force for the formation of such hydrogen bonds. Concomitantly with the formation of the activated complex, Asp23^Trx^ moves 10 Å away from Cys32^Trx^, arguably too far away to deprotonate Cys32^Trx^. Indeed, mutating Asp23^Trx^ to Ala cannot prevent complex dissociation (Figure 8B). This experiment demonstrates that Asp23^Trx^ is not critical for activation of Cys32^Trx^. The same was found for the putative deprotonation role of Cys82^ArsC^. In the absence of Cys82^ArsC^, the mixed disulfide complex still dissociates. So, the transient hydrogen bonding of Cys32^Trx^S_γ_ on the timescale explored during the MD simulations is enough to stabilize Cys32^Trx^ as a nucleophilic thiolate, leading to the reduction of the disulfide bond in its substrate.

Hydrogen bonding by Arg16, Arg108, Thr11 and Gly12 will drop the pKa of Cys82^ArsC^ to 6.3 (Figure S1 in Supplemental data). Under physiological conditions, not only Cys82^ArsC^ but also Cys32^Trx^ (pKa = 7.4) predominates as a thiolate, due to two hydrogen bonds to its sulphur. Thiols with pKa values close to but lower than the pH of the solution react most rapidly [55], indicating that Cys82^ArsC^ would be more reactive than Cys32^Trx^. However, Cys82^ArsC^ never approaches the mixed disulfide during the MD simulations. Further, reactivity analysis predicts that Cys32^Trx^ is less soft than Cys82^ArsC^ and thus more prone to nucleophilically attack the less soft Cys29^Trx^-Cys89^ArsC^ disulfide.

During the Cys29^Trx^-Cys89^ArsC^ complex dissociation, Cys29^Trx^ or Cys89^ArsC^ might be the leaving group of the thiol/disulfide exchange reaction (Figure 2). The calculated pKa of Cys89^ArsC^ at the C-terminal of the partially unfolded redox helix in Sa_ArsC is 6.7 [42], while the calculated pKa of Cys29^Trx^ in Bs_Trx is 5.5 (Table 1). Based solely on the negative correlation between the rate of a thiol-disulfide exchange and the pKa of the leaving thiol [51]–[54], the rate with Cys29^Trx^ is expected to be higher than with Cys89^ArsC^ as leaving group. However, Cys89^ArsC^ is the leaving group. More important is the structure argument in which a thiolate attacks a disulfide bond preferentially along the sulphur-sulphur axis [56],[57] favoring the attack of Cys32^Trx^ on Cys29^Trx^. Further, we calculated that Cys29^Trx^ in the Cys29^Trx^-Cys89^ArsC^ disulfide has a higher intrinsic reactivity than Cys89^ArsC^ (Table 5). As such, the regioselectivity analysis emerged as consistent with other, independent, structural arguments, further strengthening the reactivity analysis. Thus, the correlation between the rate of a thiol-disulfide exchange and the pKa's of the reacting and leaving thiols is only one aspect to consider and is insufficient to interpret the regioselectivity of a nucleophilic attack.

The used models for the pKa and reactivity analysis include all hydrogen bond interacting residues with the considered cysteine. The MD, pKa and reactivity calculations are performed with state-of-the-art protocols which have proved their worth in a variety of contexts [29]–[33]. The theory of chemical reactivity is well established and widely used to study generalized acid/base reactions - including most of the organic reactions (additions, substitutions, eliminations) and the inorganic complexation reactions - of which thiol-exchange reactions are an example. The application of MD simulations, pKa and reactivity calculations and experimental studies yields a consistent picture of the studied mechanism, which at the very least indicates that the choice of methods is sound. Therefore, we argue that the results presented are stronger than just a plausible hypothesis. Proving chemical mechanisms is always very difficult and proceeds via successively improved working hypotheses. Thus, the present work cannot claim to have achieved a final proof. Yet, our proposed mechanism is grounded in a credible new structural model. It allows the exclusion of some currently proposed hypotheses, and crucially, it has explanatory power regarding regioselectivity, the activation of Cys32^Trx^ and the lack of direct role for Asp23^Trx^. Therefore, the evidence gathered here arguably offers currently the most convincing and operational working model of the thiol/disulfide exchange reactions catalyzed by thioredoxin.

To give fresh insights in the experimentally observed regioselectivity of Trx-ArsC complex dissociation, information from the MD structural analysis combined with the local softness used in the context of the HSAB principle, has provided a more complete and accurate picture. Equally important, we have demonstrated a methodology of general interest. MD simulations combined with pKa calculations, reactivity analysis and biochemical experiments offer a general and powerful strategy to study thiol/disulfide exchange reactions.

## Materials and Methods

### Models of the Bs_Trx, Bs_ArsC and B. subtilis Trx-ArsC complex for DFT calculations

The linear correlation between the pKa of cysteines of different Trx and ArsC systems and the Natural Population Analysis (NPA) charge on its S_γ_-atom is obtained as described [37] (Table S1). For the used Trx and ArsC models, see Supplementary Data (legend Table S1 and Figure S3).

The DFT calculations are performed with Bs_Trx and Bs_ArsC, while all experimental work is executed with Sa_Trx and Sa_ArsC, since no structures of reduced Sa_Trx or Sa_Trx-ArsC complex are available. Bs_ArsC and Sa_ArsC are structurally very similar [16],[18],[58] and they use a similar thioredoxin-coupled reaction mechanism [13], [16]–[18]. Model systems of reduced Bs_Trx (2GZY) [58], oxidized Bs_ArsC (1Z2E) [18] and of the Bs_Trx C32S – Bs_ArsC C10S/C15A/C82S complex (2IPA) [34] are made (Figure 4) for the pKa and reactivity analysis. They contain all residues that hydrogen bond interact with the considered cysteine residues. The protein environment was modeled by a continuum solvent model with a di-electric constant of 20 [59]. See Supplemental data (Text S1) for details. To calculate the pKa of the activated Trx-ArsC complex, different MD snapshots in which Cys32-SG makes “good” hydrogen bonds to Trp28-NH and Cys29-NH were used. Good hydrogen bonds were defined as follows: SG…N distance<4 Å and SG…H-N angle>150 deg, with both Trp28-NH and Cys29-NH. A model similar to the Trx_ArsC_2_Cys32 model was made.

In all models, hydrogen atoms are placed and optimized together with the Sγ atoms of the reduced cysteine residues at the B3LYP/6-31G* level. Starting from these optimized geometries which are shown in Figure 4, NPA [41] charges for pKa estimations are calculated at the B3LYP/6-31+G** level in a continuum solvent model (PCM) with a di-electric constant of 20 [59]. Bondi radii are used to construct the cavity. An electrostatic model in which the optimized structures are represented as ChelpG point charges [42],[60],[61] was used as an approximation for the enzymatic environment, when the Fukui function and the (local) softness of Cys82, Cys32 and the Cys29-Cys89 mixed disulfide in Trx_ArsC_1 and Trx_ArsC_2, Cys29 in Trx_red and the Cys82-Cys89 disulfide in ArsC_ox are calculated. This procedure is necessary since the effect of taking one electron out or adding one electron to the system (needed to obtain the Fukui function) on the NPA charge is not sizeable in a hundred atoms large system. The ChelpG charges are calculated at B3LYP/6-31G**. Fukui functions and softnesses are calculated at the B3LYP/6-31+G** level as described in the Supplemental data (Text S2). All calculations are performed using the GAUSSIAN 03 package [62].

### Molecular dynamics simulations

Energy minimizations and MD simulations of the Bs_Trx-ArsC mixed-disulfide complex were performed with the program CHARMM [63], the version 22 of its protein force-field [64],[65], a dielectric constant of 1.0, and atom-based non-bonded interactions truncated beyond 12 Å with force-shift [66]. There is ample evidence that the 12 Å force-shift spherical cut-off used in the present work performs well, and as well as alternative PME schemes also in use currently. This is evidenced by detailed studies, which compared spherical cutoffs to PME and concluded that both schemes work equally well [67]–[69]. In addition, a recent study of a DNA oligomer in solution obtained very similar results with either CHARMM and the force-shift cutoff or AMBER and PME [70]. Also, the 12 Å force-shift spherical cut-off was used across a series of studies on proteins in the Trx superfamily. These studies characterized the structure, dynamics and pKa's of the ionized active-site cysteines, alongside detailed comparisons to experimental observables [31]–[33]. In every case, excellent agreement was found between simulations and experiment, even at predictive level. This is a very strong validation of the 12 Å force-shift spherical cut-off. Therefore, we continue to use this cutoff in the present work for reasons of consistency and to allow for comparisons across different proteins of the Trx superfamily. Non-bonded lists were maintained to 14 Å and updated heuristically.

The initial coordinates were from conformer 7 of the NMR structure (2IPA). To stabilize this complex for NMR work, Cys32^Trx^, Cys10^ArsC^ and Cys82^ArsC^ had been mutated to Ser residues, and Cys15^ArsC^ to Ala. For the simulations, these residues were restored to their wild type sequence by a straightforward modeling operation. Then, two complexes were setup, with Cys82^ArsC^ neutral or as a thiolate. Force-field parameters for the thiolate were as described previously [31]. Conventional protonation states were assigned to other titratable residues.

The complexes were overlaid with a rhombic dodecahedron of pre-equilibrated CHARMM TIP3P water [71],[72], with a normal distance of 88 Å between opposing faces of the dodecahedron. The protein did not see its image during the simulation since the shortest distance between the protein in the primary cell and its image was initially 21 Å, which is much larger than the longest cut-off used in the calculations (12 Å). The water molecules overlapping with the protein were removed. The systems were neutralized by the addition of sodium ions. Periodic boundary conditions were applied, and all covalent bonds involving a hydrogen were constrained with SHAKE [73]. The system was energy minimized in several stages, with the solute initially fixed and finally allowed to fully relax. The system was then submitted to MD simulations, using the leap-frog integrator, a 0.002 ps timestep, and the NPT ensemble. One MD simulation per complex (Cys82^ArsC^ neutral or thiolate) was performed. Heating was performed from 50 K to 300 K in 5ps by 5 K increments, with the protein atoms harmonically constrained to their initial position with a force constant of 2.0 kcal/mol/Å^2^. The constraints on the solute were kept for a further 20 ps of equilibration. Then, the simulations were pursued at 300 K for at least 75 ns with each system.

### Preparation of the mixed disulfide between Sa_ArsC^Trip^ and wild type Sa_Trx and Sa_Trx D23A

Details on the Site-directed mutagenesis, expression and purification of Sa_Trx and Sa_ArsC can be found in Supplemental Data (Text S3). An aliquot of purified Sa_ArsC^Trip^ was incubated with 20 mM DTT for 30 min at room temperature to assure that the thiol was fully reduced. The excess of DTT was removed on a Superdex75 HR (10/30) column equilibrated in poly buffer A, containing 10 mM Na-borate, 10 mM Na-phosphate, 10 mM Na-citrate.

The fractions containing ArsC were pooled, concentrated and 10 mM DTNB was added. The reaction was monitored at 412 nm for the completion of the reaction. The reaction mixture was applied a second time to the gel filtration column to remove excess DTNB and released TNB^−^. The fraction of mixed disulfide Sa_ArsC^Trip^–TNB was concentrated and used directly in the reaction with wild type or Sa_Trx D23A.

Wild type and D23A Sa_Trx were incubated with 20 mM fresh DTT at room temperature for 30 minutes to assure that the remaining active site thiol was in the reduced state. The excess of DTT was removed on a superdex75 HR (10/30) column equilibrated in poly buffer A.

Wild type and Sa_Trx C32A were reacted with an equimolar concentration of Sa_ArsC^Trip^ at room temperature. Upon completion of the reaction, the mixture was loaded on a Superdex75 HR (10/30) column equilibrated in poly buffer A. Complex formation was evaluated on a non-reducing SDS-PAGE and on reversed phase. The reaction mixture was injected on a Grace Vydac C18 column (4.6 mm×250 mm) equilibrated in 15% (v/v) acetonitrile, 0.1% (v/v) trifluoroacetic acid (TFA) at 0.8 ml/min. The column was eluted with a 30 min linear gradient from 15% to 60% acetonitrile at room temperature. Absorption data collection at 280 nm was performed under Empower (Waters, Milford Massachusetts).

## Supporting Information

Text S1 Models of the Bs_Trx, Bs_ArsC and B. subtilis Trx-ArsC complex for DFT calculations
(0.03 MB DOC)

Text S2 Description of reactivity
(0.03 MB DOC)

Text S3 Site-directed mutagenesis, expression and purification of Sa_Trx and Sa_ArsC
(0.03 MB DOC)

Figure S1 Hydrogen bonds formed with Arg16
(0.38 MB DOC)

Figure S2 Reaction scheme of complex formation via a TNB-mixed disulfide
(0.16 MB TIF)

Figure S3 Model system of the Trx systems
(7.08 MB TIF)

Figure S4 Determination of the pKa of Cys89 of Sa_ArsC C10S/C15A/C82A and of Cys10 of oxidized Sa_ArsC C15A
(0.10 MB DOC)

Table S1 Calculated and experimentally obtained pKas of different Trx and ArsC molecules.
(0.05 MB DOC)
