# Supplementary material for: How Thioredoxin Dissociates Its Mixed Disulfide
Source: PLoS Comput Biol. 2009 Aug 13;5(8):e1000461. doi: 10.1371/journal.pcbi.1000461 (PMC2714181; doi:10.1371/journal.pcbi.1000461)
Supplement: Text S1 — Models of the Bs_Trx, Bs_ArsC and B. subtilis Trx-ArsC complex for DFT calculations (0.03 MB DOC) [file pcbi.1000461.s001.doc]

### Text S1:

### Models of the Bs_Trx, Bs_ArsC and B. subtilis Trx-ArsC complex for DFT calculations

Reduced Bs_Trx (2GZY)^1^ is modelled by the Trp28-Cys32 active site and the adjacent α1-helix (Lys33-Glu45) (Figure 4A, Trx_red). In Trx_red_Cys29, Cys29 is deprotonated and in Trx_red_Cys32, both Cys29 and Cys32 are deprotonated.

Oxidized Bs_ArsC (1Z2E)^2^ is represented by the complete looped-out redox helix from Cys82 to Cys89 (Figure 4B, ArsC_ox). Reduced Bs_ArsC (1Z2D)^2^ is modelled by the redox helix extending from Cys82 to Cys89, Arg16 and Thr11 and is called ArsC_red.

The structure of the Bs_Trx C32S – Bs_ArsC C10S/C15A/C82S complex (2IPA)^3^ is modelled by a first model system, extracted from conformer 7 of the NMR bundle (Figure 4C, Trx_ArsC_1), including the Trx active site (Trx28-Cys32) and the ArsC redox helix (Ser82-Cys89) and Thr11. In a second model, extracted from conformer 1 of the NMR bundle (Figure 4D, Trx_ArsC_2), all structural elements present in Trx_ArsC_1 and Arg16 of ArsC are taken. In Trx_ArsC_1 and Trx_ArsC_2, Ser32^Trx^ and Ser82^ArsC^ are mutated *in silico* to Cys32^Trx^ and Cys82^ArsC^ by replacing Oγ by Sγ and adapting the Sγ-Cβ distance. The Arg16^ArsC^Ala mutant of Trx_ArsC_2 is built ‘*in silico*’ starting from the coordinates of Trx_ArsC_2_Cys82. In Trx_ArsC_1_Cys32 and Trx_ArsC_1_Cys82, Cys32 and Cys82 are respectively deprotonated. In Trx_ArsC_2_Cys82, Cys82 is deprotonated and in Trx_ArsC_2_Cys32, both Cys32 and Cys82 are deprotonated.

The Trx_ArsC_2_trunc and Trx_ArsC_1_trunc models include the Trp28-Cys32 part of Trx and Cys89 of the ArsC part (Figure 4E). Here, Cys32 is deprotonated. The influence of the Trx α-helix (Lys33-Glu45) is investigated using the ‘Trx_ArsC_1_trunc + helix’ and ‘Trx_ArsC_2_trunc + helix’ models (Figure 4F).

In all models, hydrogen atoms are placed and optimized together with the Sγ atoms of the reduced cysteine residues at the B3LYP/6-31G* level.

1. Xu, H.

The solution structure of Bacillus subtilis thioredoxin in the reduced and oxidized forms, to be published.
2. Guo, X., Li, Y., Peng, K., Hu, Y., Li, C., Xia, B., and Jin, C. (2005). Solution structures and backbone dynamics of arsenate reductase from *Bacillus subtilis*: reversible conformational switch associated with arsenate reduction.

J. Biol. Chem. *280*, 39601-39608.
3. Li, Y., Zhang, X., Xu, H., Lescop, E., Xia, B., and Jin, C. (2007). Conformational fluctuations coupled to the thiol-disulfide transfer between thioredoxin and arsenate reductase in *Bacillus subtilis*. J. Biol. Chem. *282*, 11078-11083.
