# Supplementary material for: How Thioredoxin Dissociates Its Mixed Disulfide
Source: PLoS Comput Biol. 2009 Aug 13;5(8):e1000461. doi: 10.1371/journal.pcbi.1000461 (PMC2714181; doi:10.1371/journal.pcbi.1000461)
Supplement: Text S2 — Description of reactivity (0.03 MB DOC) [file pcbi.1000461.s002.doc]

## Description of reactivity

According to the HSAB principle^1^ in its local form^2^, the preferred reactivity between two reaction partners can be extracted from the difference in local softness (*s*) of the interacting sulfur atoms (*s^+^* and *s^-^* for the electrophilic and the nucleophilic sulfur atom respectively), which should be minimal for optimal interaction (softness matching procedure).

$\Delta s = \left|s_{A}^{-} -s_{A}^{+}\right|$ (1)

The local softness *s* is obtained as

$s_{A}^{-} = Sf_{A}^{-}$ (2)

$s_{A}^{+} = Sf_{A}^{+}$ (3)

*S* is the global softness of the system^3^ and is given as^4^:

$S \approx\frac{1}{\varepsilon_{LUMO} - \varepsilon_{HOMO}}$ (4)

with $\varepsilon_{HOMO}$ the energy of the highest occupied molecular orbital and $\varepsilon_{LUMO}$ the energy of the lowest unoccupied molecular orbital.

*f* is the Fukui function^3^, condensed to atoms given as:

(Modeling the attack of an electrophile) (5)

(Modeling the attack of a nucleophile) (6)

*q_A_(N_0_), q_A_(N_0_+1)* and *q_A_(N_0_-1)* are the atomic electron populations (in this work, the Natural Population Analysis (NPA) is used) for atom A in the reference molecule (*N_0_* electrons) and the corresponding anion (*N_0_* + 1) or cation (*N_0_* - 1), all evaluated at the molecular geometry of the reference system, as required by the constant external potential ν(***r***).

###

1. Geerlings, P., De Proft, F., and Langenaeker, W. (2003). Conceptual density functional theory. Chem. Rev. *103*, 1793-1874.
2. Yang, W., Parr, R. G. (1985). Hardness, softness, and the Fukui function in the electronic theory of metals and catalysis. Proc. Natl. Acad. Sci. U.S.A. *82*, 6723-6726.
3. Damoun, S., Van dewoude, G., Mendez, F., and Geerlings, P. (1997). Local Softness as a Regioselectivity Indicator in [4+2] Cycloaddition Reactions. J. Phys. Chem. A *101*,886-893.
4. Roos, G., Loverix, S., De Proft, F., Wyns, L. and Geerlings, P. (2003). A computational and conceptual DFT study of the reactivity of anionic compounds: implications for enzymatic catalysis. J. Phys. Chem. A *107*, 6828-6836.
