# Supplementary material for: How Thioredoxin Dissociates Its Mixed Disulfide
Source: PLoS Comput Biol. 2009 Aug 13;5(8):e1000461. doi: 10.1371/journal.pcbi.1000461 (PMC2714181; doi:10.1371/journal.pcbi.1000461)
Supplement: Text S3 — Site-directed mutagenesis, expression and purification of Sa_Trx and Sa_ArsC (0.03 MB DOC) [file pcbi.1000461.s003.doc]

### Site-directed mutagenesis, expression and purification of Sa_Trx and Sa_ArsC

Wild type Sa_Trx was constructed, expressed and purified as previously described^1^ A pET-14b vector containing the wild type *trxA* gene^2^ was used as DNA template in a QuickChange site directed Mutagenesis kit (Stratagene, La Jolla, California USA) to construct the D23A mutation. The resulting pET-14b vector (Novagen, Madison, WI) was transformed in *E. coli* strain BL21 (AI) (Invitrogen, Paisley, UK). The strain was grown in a Luria-Bertani broth preculture with 100 µg/ml ampicillin at 37 °C. The culture was transferred to Terrific broth and was grown for 4 h at 37 °C with 100 µg/ml ampicillin. Induction at a cell density of approximately 0.9 was carried out overnight with 1mM IPTG and 0.2% arabinose at 28 °C. D23A Sa_Trx was purified as described for wild type Sa_Trx^15^. The Sa_ArsC C10S/C15A/C82A (Sa_ArsC^Trip^) mutation was constructed and purified as described^1,12^ for Sa_ArsC C10S/C15A/C82S.

1. Messens, J., Van Molle, I., Vanhaesebrouck, P., Limbourg, M., Van Belle, K., Wahni, K., Martins, J. C., Loris, R., and Wyns, L. (2004). How thioredoxin can reduce a buried disulfide bond. J. Mol. Biol. *339*, 527-537.
2. Messens, J., Martins, J. C., Brosens, E., Van Belle, K., Jacobs, D. M., Willem, R., and Wyns, L. (2002). Kinetics and active site dynamics of *Staphylococcus aureus* arsenate reductase. J. Biol. Inorg. Chem. *7*, 146-156.
