# Supplementary material for: How Thioredoxin Dissociates Its Mixed Disulfide
Source: PLoS Comput Biol. 2009 Aug 13;5(8):e1000461. doi: 10.1371/journal.pcbi.1000461 (PMC2714181; doi:10.1371/journal.pcbi.1000461)

**Figure S1:** Hydrogen bonds formed with Arg16

**A**. Time course in the MD simulation of the distance between Cys82^ArsC^ and Arg108^ArsC^, Arg16^ArsC^ and Thr11^ArsC^. The MD simulations are performed with ionized Cys82^ArsC^. **B.** Visualisation of the hydrogen bonds formed with Cys82^ArsC^ in the Trx-ArsC mixed disulfide.

A.
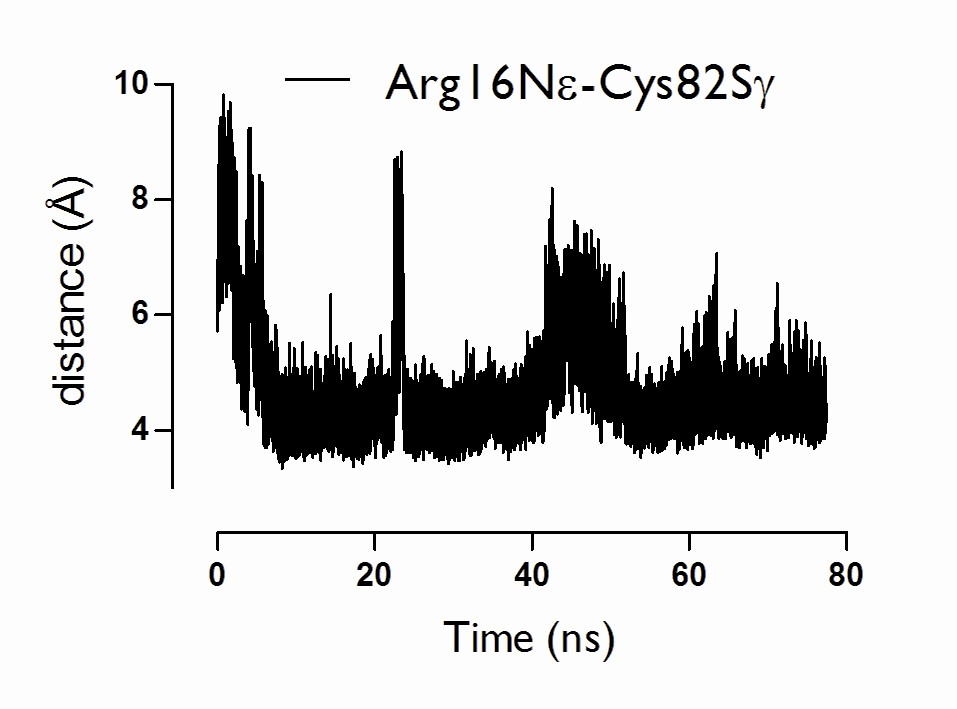

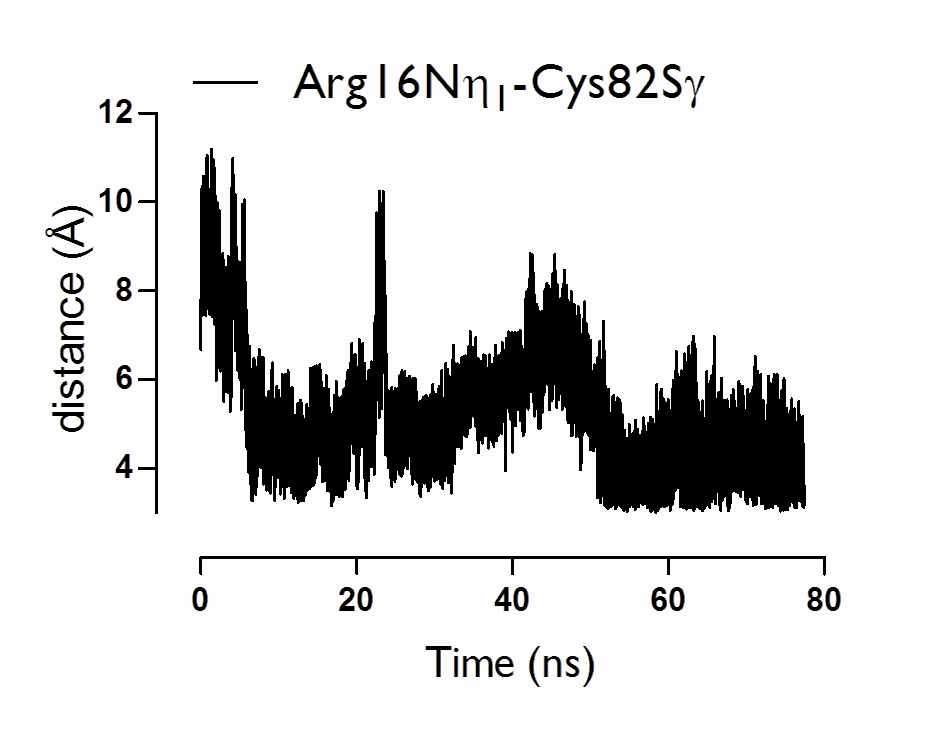


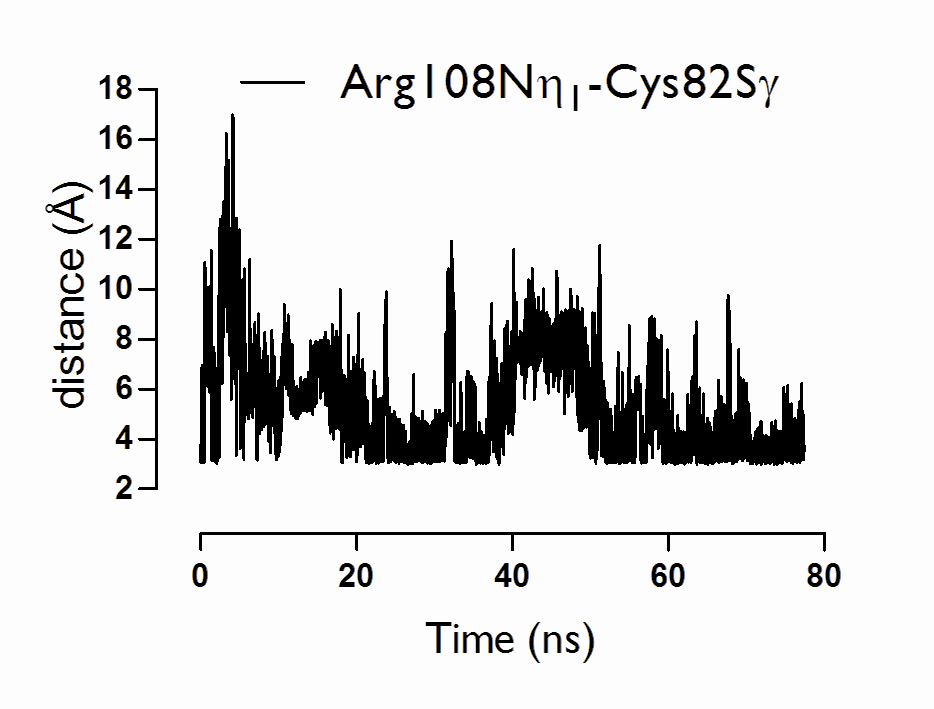

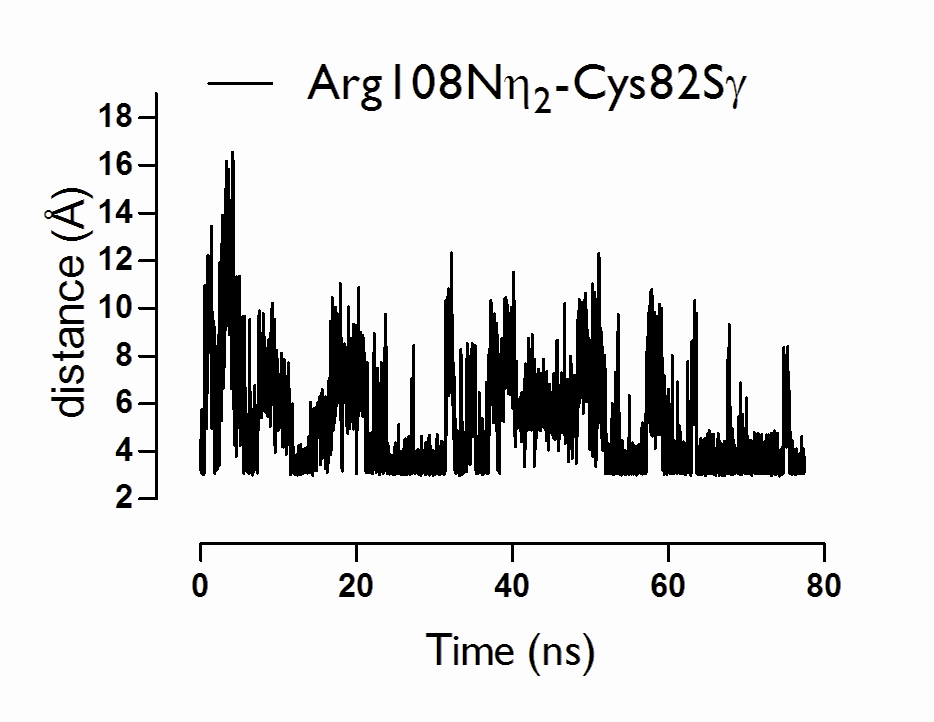


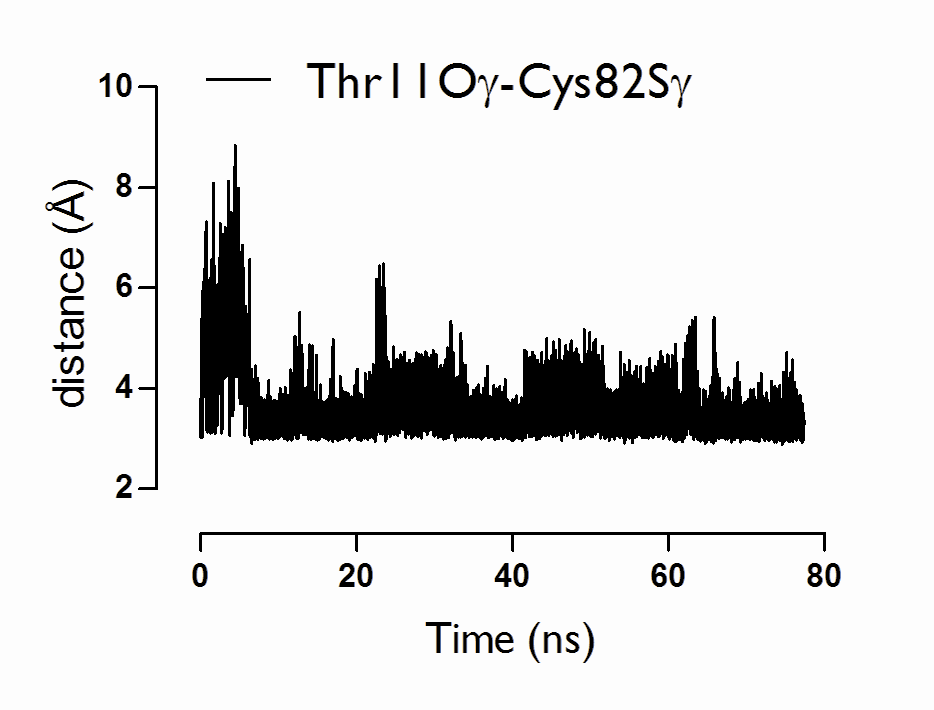

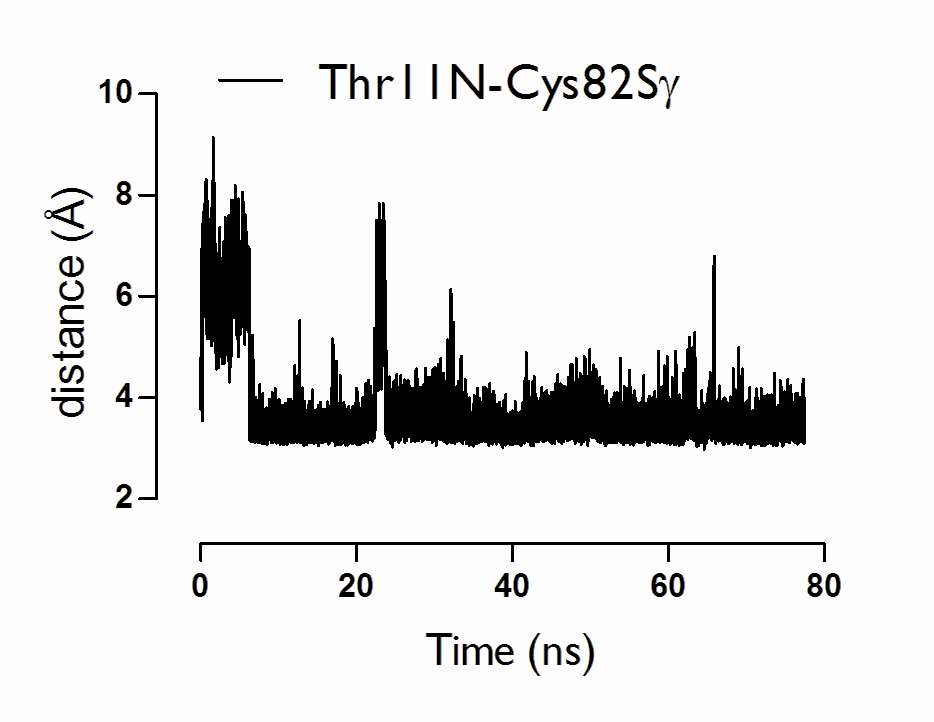


**B.**


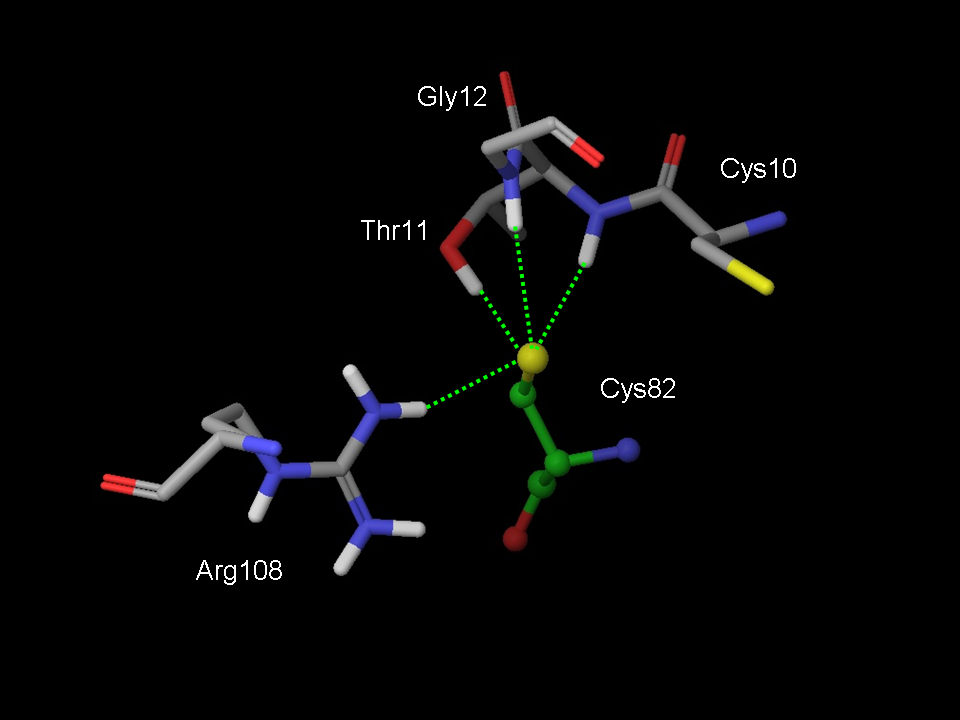

Supplement: Figure S1 — Hydrogen bonds formed with Arg16 (0.38 MB DOC) [file pcbi.1000461.s004.doc]
