# Supplementary figures and images for: How Thioredoxin Dissociates Its Mixed Disulfide
Source: PLoS Comput Biol. 2009 Aug 13;5(8):e1000461. doi: 10.1371/journal.pcbi.1000461 (PMC2714181; doi:10.1371/journal.pcbi.1000461)

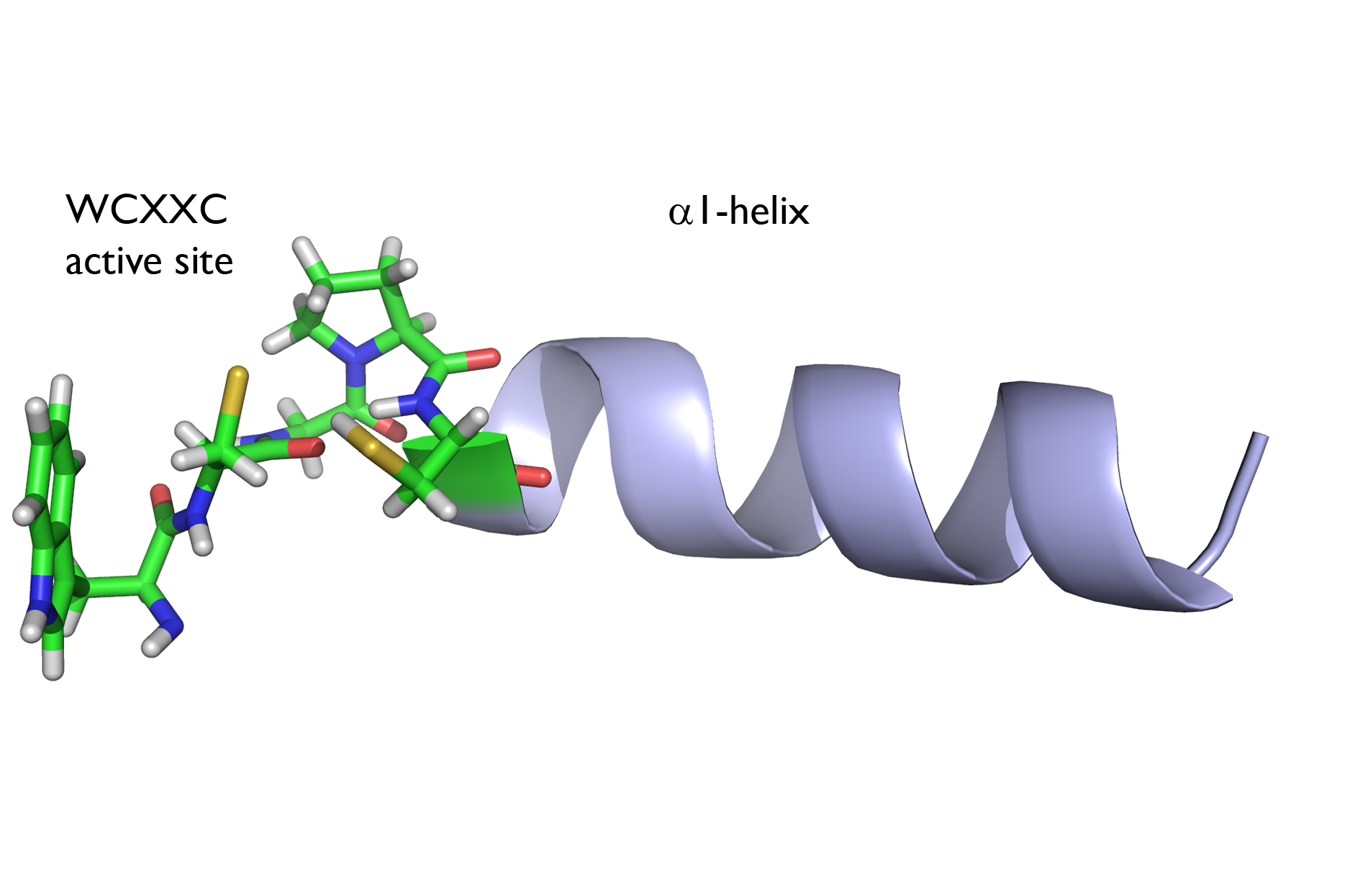

Supplement: Figure S3 — Model system of the Trx systems (7.08 MB TIF) [file pcbi.1000461.s006.tif]
