# Supplementary material for: How Thioredoxin Dissociates Its Mixed Disulfide
Source: PLoS Comput Biol. 2009 Aug 13;5(8):e1000461. doi: 10.1371/journal.pcbi.1000461 (PMC2714181; doi:10.1371/journal.pcbi.1000461)
Supplement: Figure S4 — Determination of the pKa of Cys89 of Sa_ArsC C10S/C15A/C82A and of Cys10 of oxidized Sa_ArsC C15A (0.10 MB DOC) [file pcbi.1000461.s007.doc]

**Figure S4:** **Determination of the pKa of Cys89 (A) of Sa_ArsC C10S/C15A/C82A and of Cys10 (B) of oxidized Sa_ArsC C15A**. The specific absorption of the thiolate ion at 240 nm is shown as a function of the pH.


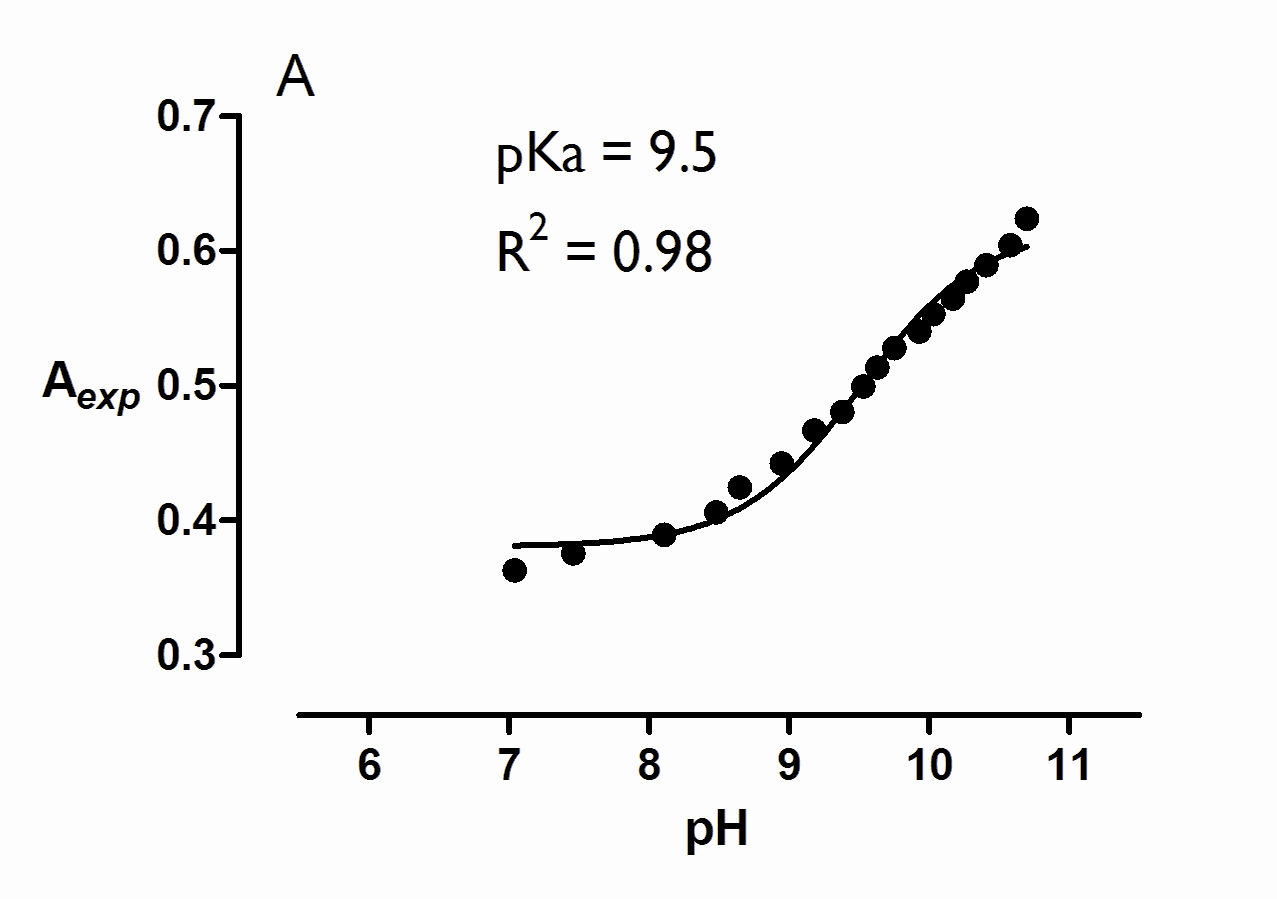


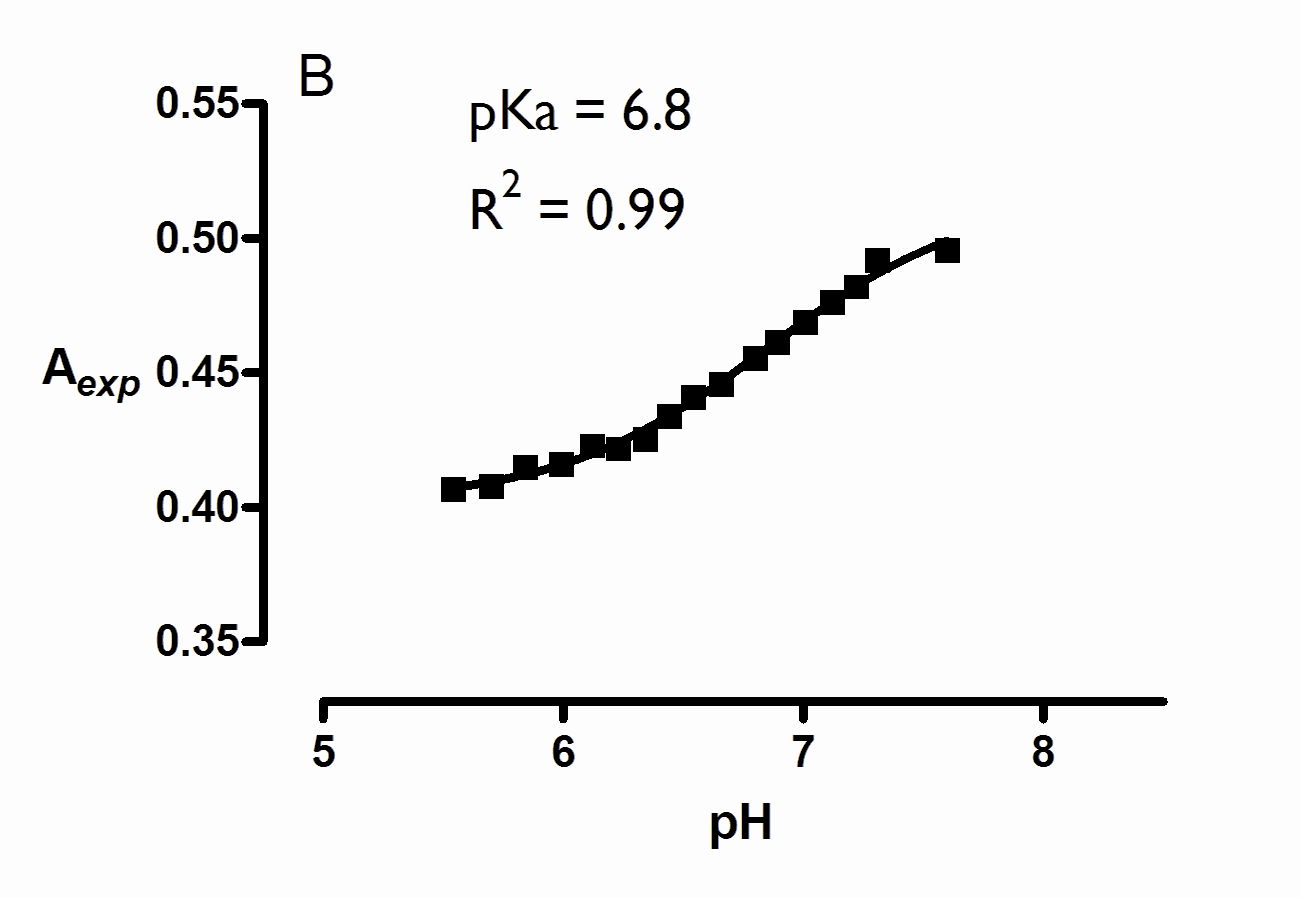


C15A Sa_ArsC was incubated with 1M arsenate at room temperature for 30 minutes to oxidize Sa_ArsC. The excess of arsenate was removed on a superdex75 HR (10/30) column equilibrated in a poly buffer containing 1mM EDTA,10 mM Na-borate, 10 mM Na-phosphate, 10 mM Na-citrate (pH 8).

An aliquot of purified C10S C15A C82S Sa_ArsC was incubated with 20mM DTT for 30 minutes at room temperature to assure that the thiol was fully reduced. The excess of DTT was removed on a Superdex75 HR (10/30) column equilibrated in a poly buffer containing 1mM EDTA, 10 mM Na-borate, 10 mM Na-phosphate, 10 mM Na-citrate and 10 mM CAPS (pH 11.4).

The thiolate ion has a higher absorption at 240 nm than the unionised thiolate group, allowing the determination of the thiol p*K*_a_ by monitoring UV absorption during pH titration^8^. For titration experiments, 100 mM HCl was stepwise added to the Sa_ArsC solution in portions of 20 to 50 µl. The absorbance at 240 nm and 280 nm was recorded on a Cary 100 Bio UV-visible spectrophotometer (Varian, Palo Alto, CA). The (A_240red_/A_280red_)/(A_240ox_/A_280ox_) value was used as a measure of the fraction of the Cys10 or Cys89 thiolate, with A_240ox_ and A_280ox_ measured from oxidized Sa_ArsC C15A. The pH dependent absorption was fitted according to the Henderson-Hasselbalch equation:

$$A_{\exp}=A_{SH}+\frac{(A_{S^{-}}-A_{SH})}{1+10^{(pKa-pH)}}$$

in which $A_{\exp}$ is $A_{240}/A_{280}$ for the experimental determined value, $A_{SH}$ is the $A_{240}/A_{280}$ value for the protonated form and $A_{S^{-}}$ is the $A_{240}/A_{280}$for the deprotonated form.
