# Supplementary material for: How Thioredoxin Dissociates Its Mixed Disulfide
Source: PLoS Comput Biol. 2009 Aug 13;5(8):e1000461. doi: 10.1371/journal.pcbi.1000461 (PMC2714181; doi:10.1371/journal.pcbi.1000461)
Supplement: Table S1 — Calculated and experimentally obtained pKas of different Trx and ArsC molecules. (0.05 MB DOC) [file pcbi.1000461.s008.doc]

**Table S1**: Calculated and experimentally obtained pKa’s of different Trx (see Figure S3 for the model system) and ArsC molecules. The calculated pKa values are obtained via the NPA-pKa correlation presented in Figure 3A of the main article.

The models of all Trxs include the WCPGC active site and the adjacent α1-helix. Sa_ArsC (1LJL) is represented by the redox helix region (Cys82-Cys89), and Arg16 and Thr11, to asses the pKa of Cys89. To calculate the pKa of Cys10, wild type Sa_ArsC (1LJL) is modelled by using its active site (Cys10-Ser17), the adjacent α1-helix (Gln18-Leu28) and the K^+^-binding pocket.

| **species** | **PDB** | **WCXXC**  **active site** | **α1-helix** | **Cysteine residue** | **Calculated pKa** | **Experimen-tally obtained pKa** |
| --- | --- | --- | --- | --- | --- | --- |
| *E. coli* Trx1 | 1XOB^1^ | Trp31-Cys35 | Lys36-Glu48 | Cys32 | 6.5 | 7.1 (ref. 2) |
| *S. aureus* Trx1  (P31T C32S) | 2O89^3^ | Trp28-Ser32 | Lys33-Glu45 | Cys29 | 6.5 | 6.4 (ref. 3) |
| *R. capsulatus* Trx2 | 2PPT^4^ | Trp72-Cys76 | Arg77-Gly92 | Cys73 | 4.8 | 5.2^*^ (ref. 5) |
| *B. subtilis* resA | 1SU9^6^ | Trp72-Cys77 | Glu78-Phe90 | Cys76 | 8.1 | 8.2 (ref. 6) |
| *S. aureus* ArsC | 1LJL^7^ | / | Cys82-Cys89 | Cys89 | 10.0 | 9.5^**^ |
| *S. aureus* ArsC | 1LJL^7^ | / | Gln18-Leu28 | Cys10 | 6.9 | 6.8^***^ |

^*^ pKa value obtained from *E. coli* Trx2

^**^pKa value obtained from C15A/C10S/C82A Sa_ArsC, see Figure S4A for the pH titration curve.

^***^pKa value obtained from oxidized C15A Sa_ArsC, see Figure S4B for the pH titration curve.

1. Jeng, M. F., Campbell, A. P., Begley, T., Holmgren, A., Case, D.A., Wright, P.E., and Dyson, H. J.

(1994). High-resolution solution structures of oxidized and reduced Escherichia coli thioredoxin.

Structure

*2*, 853-868.


2. Dyson, H. J., Jeng, M.-F., Tennant, L. L., Slaby, I., Lindell, M., Cui, D.-S., Kuprin, S., and Holmgren, A. (1997) Effects of buried charged groups on cysteine thiol ionization and reactivity in *Escherichia coli* thioredoxin: structural and functional characterization of mutants of Asp26 and Lys57. Biochemistry *36*, 2622-2636.
3. Roos, G., Garcia-Pino, A., Van Belle, K., Brosens, E., Wahni, K., Vandenbussche, G., Wyns, L., Loris, R., and Messens, J.

(2007). The conserved active site proline determines the reducing power of Staphylococcus aureus thioredoxin.

J. Mol. Biol.

*368*, 800-811.


4. Ye, J., Cho, S., Fuselier, J., Li, J., Beckwith, J., and Rapoport, T.

 (2007). Crystal structure of an unusual thioredoxin protein with a zinc finger domain. J. Biol. Chem. *282*, 34945-34957.
5. El Hajjaji, H., Dumoulin, M., Matagne, A., Colau, D., Roos, G., Messens, J. and Collet, J.-F. (2008). The zinc centre influences the redox and thermodynamic properties of *Escherichia coli* thioredoxin 2. J. Mol. Biol., in revision.
6. a) Crow, A., Acheson, R. M., Le Brun, N. E.,and Oubrie, A.

(2004). Structural basis of redox-coupled protein substrate selection by the cytochrome c biosynthesis protein ResA.

J. Biol. Chem.

*279*, 23654-23660.

b) Lewin, A., Crow, A., Hodson, C. T. C., Hederstedt, L., and Le Brun, N. E. (2008). Effects of substitutions in the CXXC active-site motif of the extracytoplasmic thioredoxin ResA, Biochem J. *414*, 81-91.
7. Zegers, I., Martins, J. C., Willem, R.,Wyns, L., and Messens, J. (2001). Arsenate reductase from *S. aureus* plasmid pI258 is a phosphatase drafted for redox duty. Nature Struct. Biol. *8*, 843-847.
